# Predicting loss of hepatitis B surface antigen and evaluating the durability of functional cure induced by pegylated interferon alpha: insights from a real-world study

**DOI:** 10.7717/peerj.20587

**Published:** 2026-01-21

**Authors:** Xin Xu, Jia-Quan Huang, Shuaiwen Huang

**Affiliations:** 1 Department of General Practice, Tongji Hospital, Tongji Medical College, Huazhong University of Science and Technology, WuHan, China; 2 Department and Institute of Infectious Disease, Tongji Hospital, Tongji Medical College, Huazhong University of Science and Technology, Wuhan, China; 3 Division of Nephrology, Tongji Hospital, Tongji Medical College, Huazhong University of Science and Technology, Wuhan, China; 4 Department of Nutrition, Tongji Hospital, Tongji Medical College, Huazhong University of Science and Technology, Wuhan, China

**Keywords:** Peg-IFN-α, HBV, HBsAg loss, Durability, Real-World Study, Functional cure

## Abstract

**Background:**

This study aims to identify the key factors influencing the loss of hepatitis B surface antigen (HBsAg) and durability of functional cure. Understanding these factors is crucial for identifying patient groups that may benefit from a pegylated interferon alpha (Peg-IFN-α) induced “functional cure” for hepatitis B, as well as for optimizing strategies to achieve and sustain HBsAg loss.

**Methods:**

The study utilized real-world data, including 378 patients with chronic hepatitis B virus (HBV) infection who received treatment with Peg-IFN-αfor 24 weeks or longer. Patients were grouped based on their response at 24 and 48 weeks of treatment, and predictive factors for the response were calculated through regression analysis. Additionally, 195 subjects who achieved HBsAg loss were included to assess factors influencing the durability of HBsAg loss. The primary endpoint was reverse seroconversion of HBsAg (HBsAg-RS).

**Results:**

The findings suggest that the degree of HBsAg at baseline and HBsAg decline at 12/24 weeks of treatment is critical for predicting treatment response. Factors affecting durability include age, nucleoside treatment, baseline hepatitis B core antibody (anti-HBc) levels, the consolidation of Peg-IFN-α therapy, and hepatitis B surface antibody (anti-HBs) levels at the time of treatment discontinuation. Higher anti-HBs levels at discontinuation and higher baseline anti-HBc levels were associated with a reduced risk of HBsAg-RS. Moreover, a significant linear dose-response relationship was observed between anti-HBs levels and the risk of HBsAg-RS after HBsAg loss.

**Conclusions:**

These results provide valuable insights for predicting responses to Peg-IFN-αtherapy and identifying high-risk groups for HBsAg-RS following HBsAg loss. This information may help refine strategies for achieving and maintaining HBsAg loss and guide post-HBsAg loss monitoring and management procedures.

## Introduction

The eradication of hepatitis B virus (HBV) remains a challenge due to the inability to directly target covalently closed circular DNA (cccDNA) and integrated HBV DNA in the host genome (Shi & Zheng, 2020; Tang et al., 2018). However, hepatitis B surface antigen (HBsAg) loss, recommended as the therapeutic goal for hepatitis B, represents stable immunologic control of HBV and is associated with improved clinical outcomes (Terrault et al., 2018; Yuen et al., 2008). Achieving HBsAg loss is considered a “functional cure” or “clinical cure” of hepatitis B (Lampertico et al., 2017; Terrault et al., 2018). HBsAg loss has been rare in patients with chronic hepatitis B, with reported rates ranging from 0% to 3.0% through spontaneous or nucleos(t)ide analogs (NAs) treatment-mediated loss (Chang et al., 2006; Marcellin et al., 2008). While some studies suggest that a limited course of nucleoside drug therapy may increase HBsAg loss, virologic rebound and relapse are common after discontinuation of NAs (Hirode et al., 2022; Lampertico & Berg, 2018; Wong et al., 2020). Recent clinical studies have demonstrated that an optimized treatment regimen based on pegylated interferon alpha (Peg-IFN-*α*) can significantly increase HBsAg loss in selected patients with dominant chronic hepatitis B (Bazinet et al., 2020; Hu et al., 2018; Ning et al., 2014). HBsAg loss not only implies delayed disease progression and reduced risk of cirrhosis and hepatocellular carcinoma but also eliminates the social stigma associated with being HBsAg-positive, reducing prejudice and dis-crimination.

Although a standard 48-week course of Peg-IFN-*α* treatment is recommended, extending the treatment duration has been suggested to further improve HBsAg loss (Hu et al., 2018). However, poor tolerability, high costs, and administration inconvenience have resulted in suboptimal patient adherence to Peg-IFN-*α* therapy (Schinazi et al., 2018). Some patients exhibit a poor response to Peg-IFN-*α*, while others achieve HBsAg loss in a shorter duration of treatment. Identifying the group that responds well to short-term Peg-IFN-*α* treatment can enhance treatment adherence, reduce costs, and improve treatment accessibility.

On the other hand, significant progress has been made in achieving HBsAg loss with Peg-IFN-*α* therapy, the persistence of HBV in the organism after HBsAg loss in chronic hepatitis B infection remains a concern (Shi & Zheng, 2020; Song et al., 2021). Several metrics and models have been proposed to predict reverse seroconversion of HBsAg (HBsAg-RS), but their applicability to individual patients in clinical practice is limited due to population heterogeneity. Additionally, most models lack external validation due to medium or small sample sizes. Therefore, understanding the factors influencing HBsAg-RS after HBsAg loss (durable functional cure) is crucial for developing rational monitoring procedures post-HBsAg loss.

This study aims to analyze the factors influencing the response to Peg-IFN-*α* therapy and identify patients who can achieve therapeutic response and clinical benefits with a shorter treatment course. It also investigates factors affecting durable functional cure to identify high-risk groups for relapse, providing real-world data for predicting response to Peg-IFN-*α* therapy, selecting personalized treatment regimens, determining clinical endpoints, and making monitoring decisions after HBsAg loss.

## Materials & Methods

### Ethical approval

This is a retrospective real-world study. This study was approved by the Medical Ethics Committee of Tongji Medical College, Huazhong University of Science and Technology (IRBID: 2021-S183). All procedures were conducted in accordance with the ethical guidelines established by the Medical Ethics Committee of Tongji Medical College, Huazhong University of Science and Technology, and the principles outlined in the Declaration of Helsinki.

Written informed consent was received from all participants of this study.

### Subject selection

Inclusion Criteria: Patients with chronic HBV infection attending outpatient clinics at Tongji Hospital, Tongji Medical College, Huazhong University of Science and Technology, China, were included in the study if they met the following criteria: (1) Aged between 18 and 65 years old. (2) HBsAg-positive for 6 months or more. (3) Chronic HBV-infected patients who received 24 weeks or more of treatment with Peg-IFN-*α*-2b (Xiamen Amoytop Biotech Co., LTD, Xiamen, China).

Exclusion Criteria: (1) Patients with contraindications to interferon use. (2) Patients with hepatitis C virus (HCV), human immunodeficiency virus infection, hepatocellular carcinoma, autoimmune hepatitis, or other serious underlying diseases.

**Figure 1:**
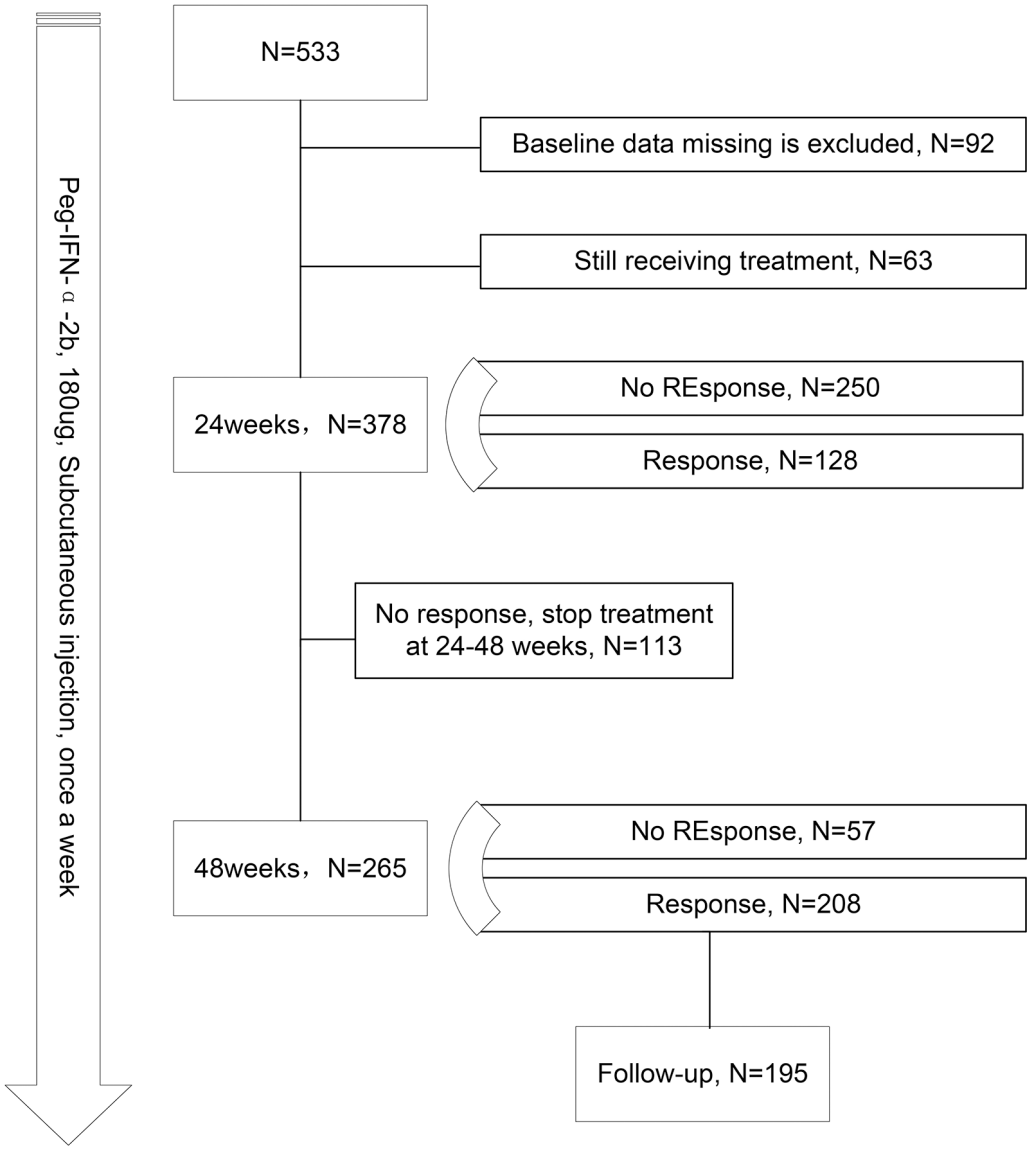
Flow chart of research.

A total of 533 patients initially met the inclusion criteria. Patients with incomplete information at baseline and those still on Peg-IFN-*α* therapy were further excluded. Ultimately, 378 patients were included in the analysis for predicting the response at 24 weeks of treatment. Among them, 113 patients discontinued Peg-IFN-*α* during 24 to 48 weeks due to treatment intolerance and cost. For the analysis of response at 48 weeks of treatment, 265 patients were included. To investigate factors affecting HBsAg-RS after HBsAg loss, 195 patients who achieved HBsAg loss through Peg-IFN-*α* therapy and underwent clinical follow-up were included (Fig. 1).

### Treatment and monitoring

All subjects received a 24-week or longer course of Peg-IFN-*α* therapy (Peg-IFN-*α*-2b, 180 ug, subcutaneous injection, once weekly). Patients with prior exposure to nucleos(t)ide analogues (NAs) continued receiving NA therapy,pegylated interferon (Peg-IFN) add-on therapy was administered to pursue functional cure. NA-naïve patients were considered for combination therapy with NAs based on a comprehensive assessment including patient age, family history, baseline HBV DNA levels, liver function markers (Alanine aminotransferase—ALT, aspartate aminotransferase—AST), and patient preference. NA-naïve patients who consent to NA therapy will initiate a combination regimen of NA and Peg-IFN.

The baseline and regular follow-up assessments included pre-treatment blood tests, liver function tests, serum hepatitis B virus (HBV) markers (quantitative hepatitis B surface antigen (HBsAg), hepatitis B e antigen (HBeAg), hepatitis B e antibody (anti-HBe), hepatitis B core antibody (anti-HBc), and hepatitis B surface antibody (anti-HBs)), hepatitis B DNA testing, and liver ultrasound. Patients were advised to undergo regular blood tests, liver function tests, Serum HBV markers, and hepatitis B DNA testing every 12 weeks or as recommended until HBsAg loss was achieved or discontinuation of Peg-IFN-*α* treatment was considered.

### Data collection and variable definition

Patient demographic information, treatment history, and blood test data were obtained from the hospital’s electronic medical record system. Serologic test data were collected at 12, 24, 36, and 48 weeks after the start of treatment. Changes from baseline in each indicator during the early stages of treatment were calculated. Nucleoside analog treatment was defined as 6 months or more of nucleoside therapy prior to the initiation of Peg-IFN-*α*. Treatment response was defined as serum HBsAg below the lower limit of detection (The quantitative level of HBsAg, measured using an electrochemiluminescence instrument (Roche), was below 0.5 ng/mL), indicating HBsAg loss at the serologic level. Serologic test information was collected for the first confirmed HBsAg loss, treatment discontinuation, and the most recent follow-up time point. HBsAg loss was defined as having two serum HBsAg results below the lower limit of detection. HBsAg-RS was defined as the redetection of HBsAg after a confirmed HBsAg loss. The diagnosis of cirrhosis was based on imaging studies and clinical diagnosis, while the diagnosis of fatty liver primarily relied on ultrasonography.

### Statistical analysis

Data with a normal distribution were presented as mean ±  standard deviation. Differences between groups were compared using appropriate statistical tests such as Student’s *t*-test, Mann–Whitney *U*-test, or one-way analysis of variance (ANOVA). Skewed distribution data were presented as median (interquartile range) and analyzed using the rank sum test. Categorical data were presented as numbers and percentages and compared using the *χ*2 test. Binary logistic regression analyses were conducted to analyze the predictors of HBsAg loss, and a logistic regression model was established. The area under the curve (AUC) of the logistic regression model was calculated, and receiver operating characteristic (ROC) curves were plotted to evaluate the predictive value of the indicators affecting the Peg-IFN-*α* response. Cumulative risk function and unifactorial and multifactorial Cox regression risk models were constructed to analyze the risk ratio of HBsAg HBsAg-RS after HBsAg loss in patients. Restricted cubic spline analysis was used to examine the associations of continuous variables with the risk of HBsAg reversal.

We randomly divided into a validation set and a training set to avoid overoptimistic internal validation. Additionally, the full dataset has been kept intact. In order to calculate the confidence interval of AUC, Our analysis utilized a bootstrap resampling approach. This method generates numerous replicate datasets by sampling with replacement, allowing for the calculation of a robust confidence interval for the AUC from the distribution of the results. This analysis was implemented using the timeROC package. Nomograms and decision curve analysis (DCA) curves were plotted using hdnom and ggDCA package in R statistical analysis software.

All statistical analyses were performed using IBM SPSS statistical analysis soft-ware (version 23.0) and R statistical analysis software (version 4.1.0; R Core Team, 2021). A significance level of *p* < 0.05 (two-tailed) was considered statistically significant.

## Results

### Analysis of predictors of achieving HBsAg loss

A total of 378 subjects underwent a 24-week Peg-IFN-*α* treatment, with 33.8% (*n* = 128) achieving HBsAg loss within this period (Fig. 1). Among the responders, 88 were males and 40 were females, with an average age of 39 years. Detailed baseline information of the subjects can be found in Table 1. Comparing responders and non-responders at the 24-week mark, it was observed that those who achieved HBsAg loss had lower baseline HBsAg and HBV DNA levels, higher quantitative anti-HBc levels, and experienced more significant reductions in HBsAg at 12 weeks of treatment.

**Table 1:** Baseline information for subjects grouped according to response to treatment at 24 weeks.

	**All**	**24W no response**	**24W response**	***P* value**
N	378	250	128	–
Gender, female(%)	112 (29.6%)	72 (28.8%)	40 (31.2%)	0.708
Age, years (mean ± SD)	39.43 ± 8.60	39.22 ± 8.97	39.85 ± 7.85	0.497
NAs (%)	198 (53.8%)	122 (50.2%)	76 (60.8%)	0.069
HBeAg (+) (%)	33 (9.1%)	32 (13.4%)	1 (0.8%)	<0.001^*^
NAFLD (%)	110 (34.8%)	77 (36.7%)	33 (31.1%)	0.395
Cirrhosis (%)	8 (2.5%)	6 (2.8%)	2 (1.9%)	0.889
Splenomegaly (%)	27 (8.5%)	20 (9.4%)	7 (6.5%)	0.507
Baselines				
HBsAg (log10 ng/mL)	2.22 ± 1.12	2.56 ± 1.00	1.55 ± 1.02	<0.001^*^
Anti-HBs (mIU/mL)	2.15 ± 16.40	1.69 ± 11.33	3.03 ± 23.27	0.457
Anti-HBe (log10 NcU/mL)	1.46 ± 0.98	1.40 ± 1.06	1.58 ± 0.77	0.097
Anti-HBc (log10 NcU/mL)	2.06 ± 0.30	2.04 ± 0.33	2.11 ± 0.24	0.030^*^
HBV DNA (log10 IU/mL)	1.19 ± 1.71	1.34 ± 1.84	0.88 ± 1.38	0.013^*^
AST (U/L)	26.74 ± 20.18	27.69 ± 23.35	24.79 ± 10.94	0.251
ALT (U/L)	31.73 ± 40.68	33.58 ± 46.66	27.93 ± 23.82	0.267
*γ*-GT (U/L)	28.40 ± 31.99	29.52 ± 35.93	26.11 ± 21.77	0.394
TBIL (umol /L)	13.38 ± 8.85	13.00 ± 7.21	14.17 ± 11.52	0.292
TP (g/L)	77.28 ± 4.08	77.32 ± 4.15	77.19 ± 3.97	0.796
12 Week				
HBsAg (log10 ng/mL)	1.32 ± 1.56	1.98 ± 1.32	−0.15 ± 0.94	<0.001^*^
Anti-HBs (mIU/mL)	4.63 ± 24.65	3.00 ± 19.04	8.29 ± 33.94	0.085
Anti-HBe (log10 NcU/mL)	1.45 ± 0.89	1.43 ± 0.92	1.49 ± 0.80	0.600
Anti-HBc (log10 NcU/mL)	2.08 ± 0.37	2.07 ± 0.40	2.09 ± 0.27	0.595
DNA (log10 IU/mL)	0.17 ± 0.62	0.24 ± 0.73	0.03 ± 0.28	<0.001^*^
AST (U/L)	66.04 ± 49.38	65.68 ± 51.76	66.86 ± 43.68	0.855
ALT (U/L)	69.58 ± 57.42	71.64 ± 61.73	64.84 ± 45.95	0.363
*γ*-GT (U/L)	76.89 ± 84.92	73.23 ± 83.75	85.44 ± 87.50	0.271
TBIL (umol /L)	11.74 ± 5.84	12.00 ± 6.41	11.14 ± 4.21	0.257
TP (g/L)	73.79 ± 4.31	73.77 ± 4.32	73.84 ± 4.33	0.895
Δ12 Week				
ΔHBsAg (log10 ng/mL)	1.00 ± 1.04	0.66 ± 0.85	1.74 ± 1.04	<0.001^*^
ΔAnti-HBs (mIU/mL)	2.24 ± 24.51	1.25 ± 11.41	4.43 ± 40.78	0.302
ΔAnti-HBc (log10 NcU/mL)	0.00 ± 0.37	0.01 ± 0.39	−0.03 ± 0.33	0.401
ΔAST (U/L)	39.46 ± 53.95	37.61 ± 56.60	43.82 ± 47.19	0.427
ΔALT (U/L)	38.67 ± 68.95	36.96 ± 74.98	42.71 ± 52.24	0.566
Δ*γ*-GT (U/L)	49.00 ± 83.62	43.78 ± 81.95	61.55 ± 86.85	0.144
ΔTBIL (umol /L)	−1.68 ± 9.02	−1.17 ± 6.80	−2.89 ± 12.86	0.190

**Notes.**

All data are expressed as number of cases (%), mean ± standard error or median (interquartile spacing).

* *P* < 0.05 indicates a statistically significant difference between the two groups.

To evaluate the characteristics and factors influencing treatment response at the 24-week mark, both univariate and multivariate logistic regression analyses were conducted (Tables S1 and S2). The results of the univariate analyses revealed that HBeAg positivity at baseline, baseline HBsAg levels, anti-HBc levels, HBV DNA levels, HBsAg levels at week 12 of treatment, HBV DNA levels at week 12 of treatment, and the change in HBsAg from baseline at 12 weeks of treatment were significant predictors of response. To assess the predictive value of metrics related to interferon response, the area under the curve (AUC) of the logistic regression model was calculated, and the ROC curve was plotted (Fig. S1). Baseline HBsAg level, anti-HBc level, HBeAg positivity, and HBV DNA level had limited predictive efficacy for short-term response. However, combining nucleoside treatment or no treatment with baseline HBsAg level, HBeAg positivity, and HBV DNA level improved the predictive efficacy (AUROC: 0.798, 95% CI [0.751–0.845]). The predictive efficacy was further enhanced when 12-week HBsAg reduction was included in the model (AUROC: 0.911, 95% CI [0.879–0.946]).

Analysis of predictive factors for achieving HBsAg loss with a standard course of treatment (48 weeks Peg-IFN-*α* treatment) were presented in the Supplementary Results, Tables S3, S4, and S5.

### Analysis of factors influencing HBsAg-RS after Peg-IFN-*α* induced HBsAg loss

**Figure 2:**
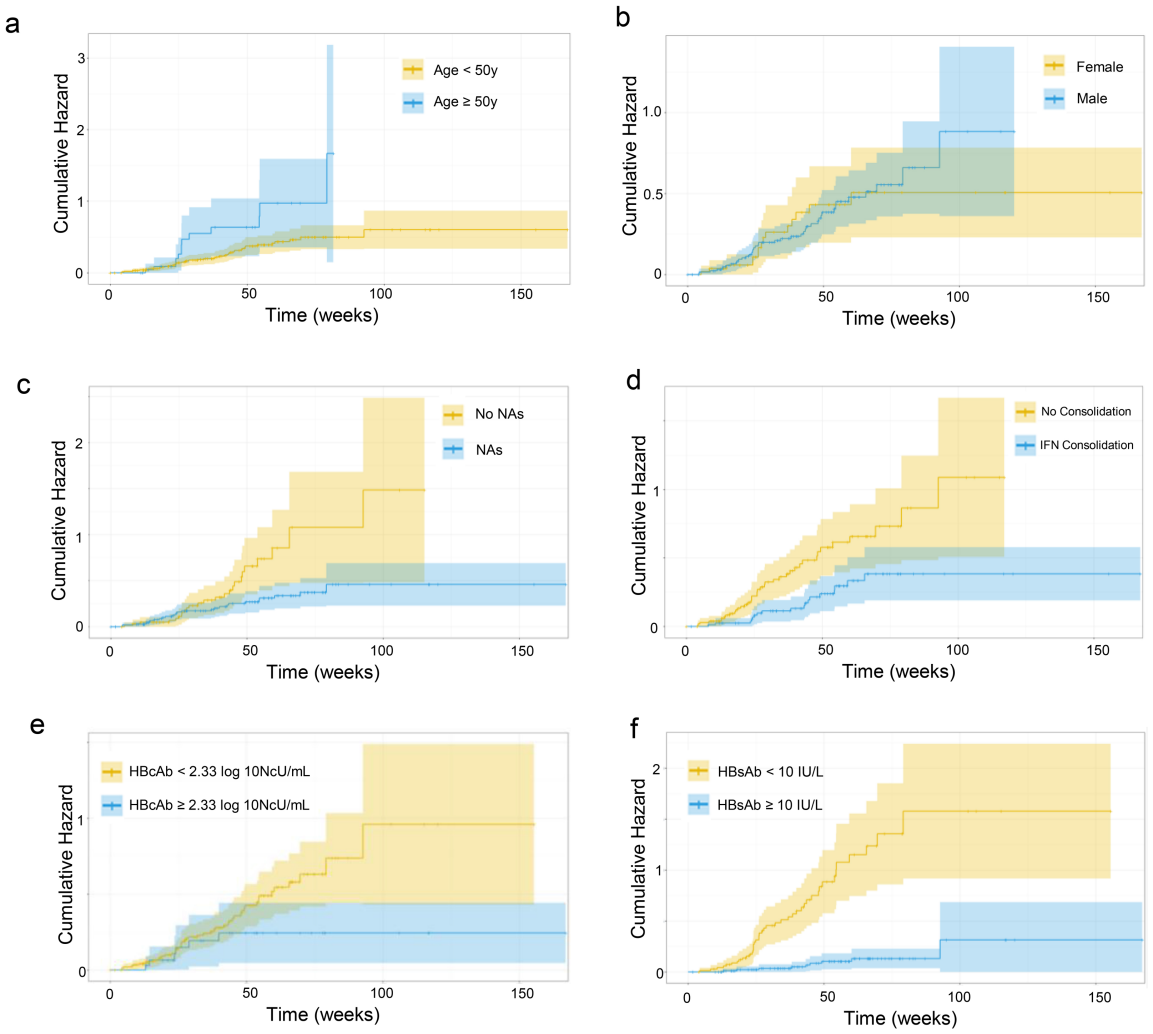
(A–F) Cumulative probability curve of HBsAg-RS.

After achieving HBsAg loss with Peg-IFN-*α* treatment, 195 subjects underwent further follow-up, and during a median follow-up of 279 days, 57 cases of HBsAg-RS occurred (cumulative incidence of 29.2%), with 36 of them occurring within 48 weeks (Table S6). The cumulative probability curves for HBsAg-RS, grouped by age, sex, baseline anti-HBc level, nucleoside treatment, duration of consolidation on Peg-IFN-*α* therapy after HBsAg loss, and anti-HBs level at the cessation of Peg-IFN-*α* therapy, are shown in Fig. 2.

Univariate and multivariate Cox regressions (stepwise method) were conducted to identify factors associated with a higher risk of HBsAg-RS. Hazard ratios (HRs) and 95% confidence intervals are presented in Table 2. Individuals younger than 50 years, nucleoside-treated patients, those with higher baseline anti-HBc levels, those with consolidation of Peg-IFN-*α* therapy for more than three months after HBsAg loss, and those with higher anti-HBs levels at the cessation of Peg-IFN-*α* therapy had a lower risk of HBsAg-RS (Fig. 2). These variables were included in the multi-factorial Cox analysis (stepwise forward method), which indicated that baseline anti-HBc level and anti-HBs level at discontinuation were significant factors influencing HBsAg-RS (Table 2).

**Table 2:** Univariate and multivariate Cox regression analysis of HBsAg-RS after Peg-IFN-*α* induced HBsAg loss.

	**Univariate Cox regression**			**Multifactor Cox regression**		
	*P*	**HR**	**95%CI**	*P*	**HR**	**95%CI**
Age (Using <50 years of age as a reference)	0.015^*^	2.157	1.158–4.016			
Gender (Using women as a reference)	0.839	1.064	0.588–1.925			
NAs (take non treatments as a reference)	0.008^*^	0.475	0.273–0.827			
Treatment baseline HBeAg positive (negative as reference)	0.718	1.298	0.315–5.324			
Length of interferon (IFN) consolidation after response (<3 months as reference)	0.003^*^	0.426	0.243–0.746			
Hepatitis B vaccination after HBsAg clearance	0.910	0.966	0.528–1.767			
Combined NAFLD	0.767	0.915	0.507–1.650			
Combined cirrhosis (no cirrhosis as reference)	0.517	0.048	0.000–464.180			
Combined splenomegaly (no splenomegaly as reference)	0.269	0.046	0.000–10.080			
**Treatment baseline serologic indicators**						
HBsAg	0.849	0.977	0.768–1.243			
Anti-HBs	0.453	0.964	0.876–1.061			
Anti-HBe	0.613	1.077	0.907–1.437			
Anti-HBc	0.072	0.503	0.237–1.064	0.001^*^	0.289	0.136–0.612
**Serologic indicators at discontinuation of Peg-IFN-*α* therapy**						
Anti-HBs	0.000^*^	0.976	0.966–0.986	0.000^*^	0.974	0.963–0.985
Anti-HBe	0.246	1.242	0.862–1.789			
Anti-HBc	0.960	0.980	0.452–2.218			

**Notes.**

* *P* < 0.05 indicates statistical significant.

**Figure 3:**
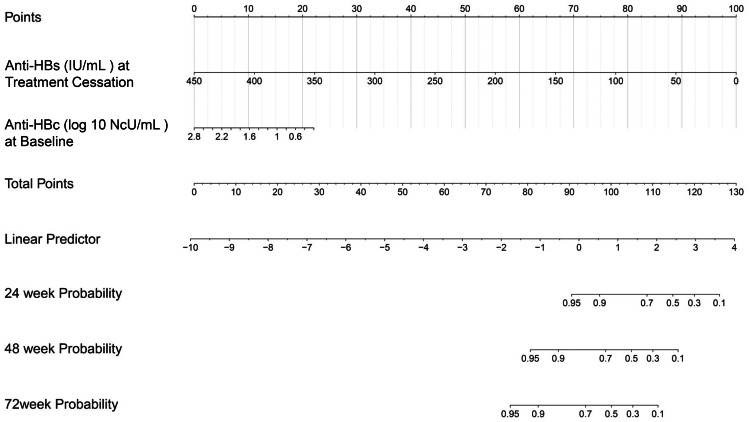
Nomogram for predicting sustained response.

**Figure 4:**
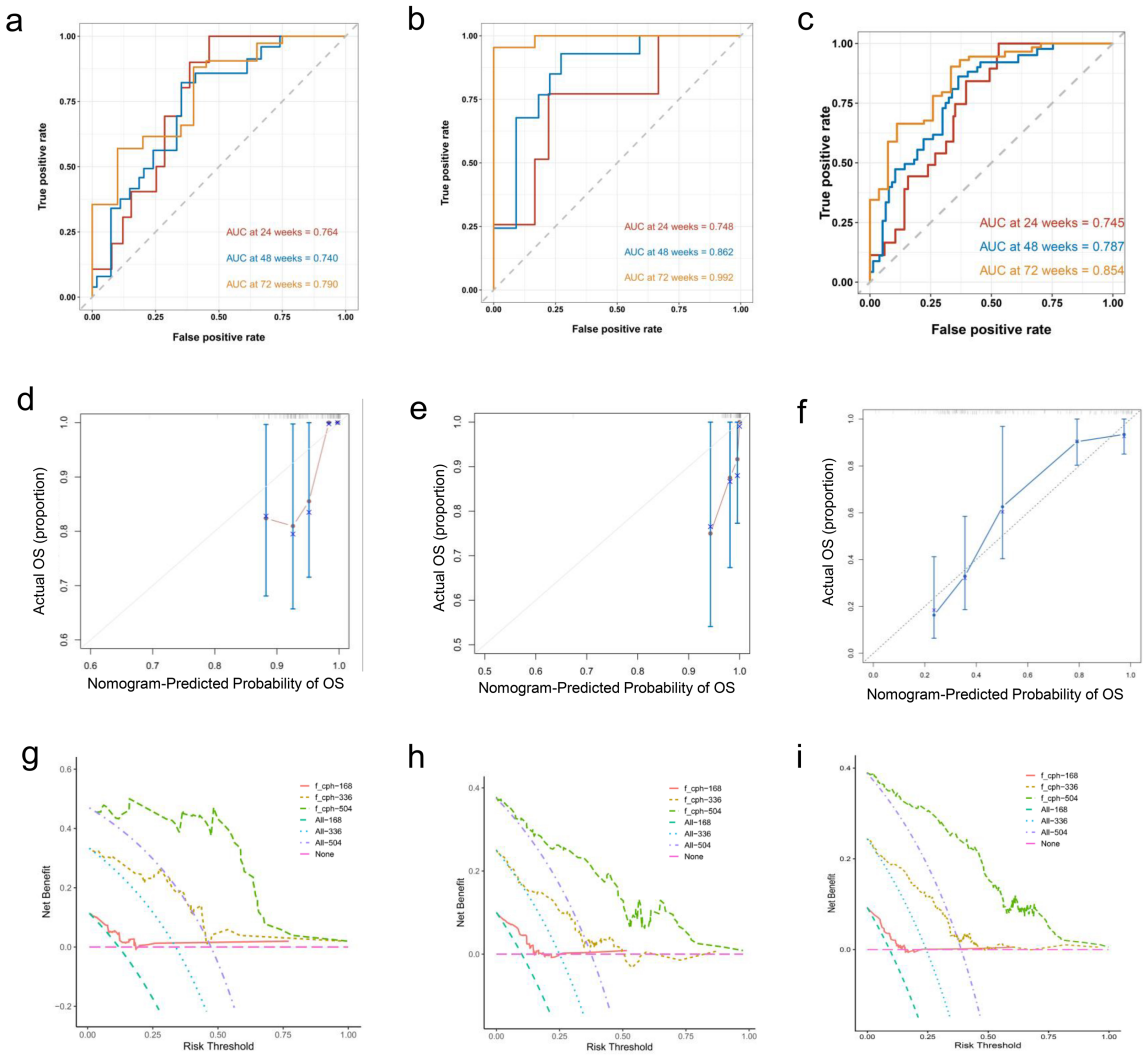
(A–I) In the training, verification and entire undivided queue, the verification results based on Bootstrap showed good robustness.

In addition, the data were divided into training and validation sets (7:3 ratio), and a Nomogram predicting the sustained response rate was developed using Cox regression based on the training set data (Fig. 3). In the training set, the AUROC for 24-week, 48-week, and 72-week sustained responders were of 0.764, 0.740, and 0.790 respectively (Fig. 4A). The corresponding AUROC values in the validation set were 0.748, 0.862, and 0.992 (Fig. 4B), while in the entire undivided dataset, they were 0.745, 0.787, and 0.854 (Fig. 4C). Bootstrap-based validation demonstrated good robustness in both the training and validation sets (Figs. 4D–4F). Additionally, decision curve analysis showed that the Nomogram had significant net benefit and a wider range of threshold probabilities in the training, validation and entire undivided dataset (Figs. 4G–4I).

**Figure 5:**
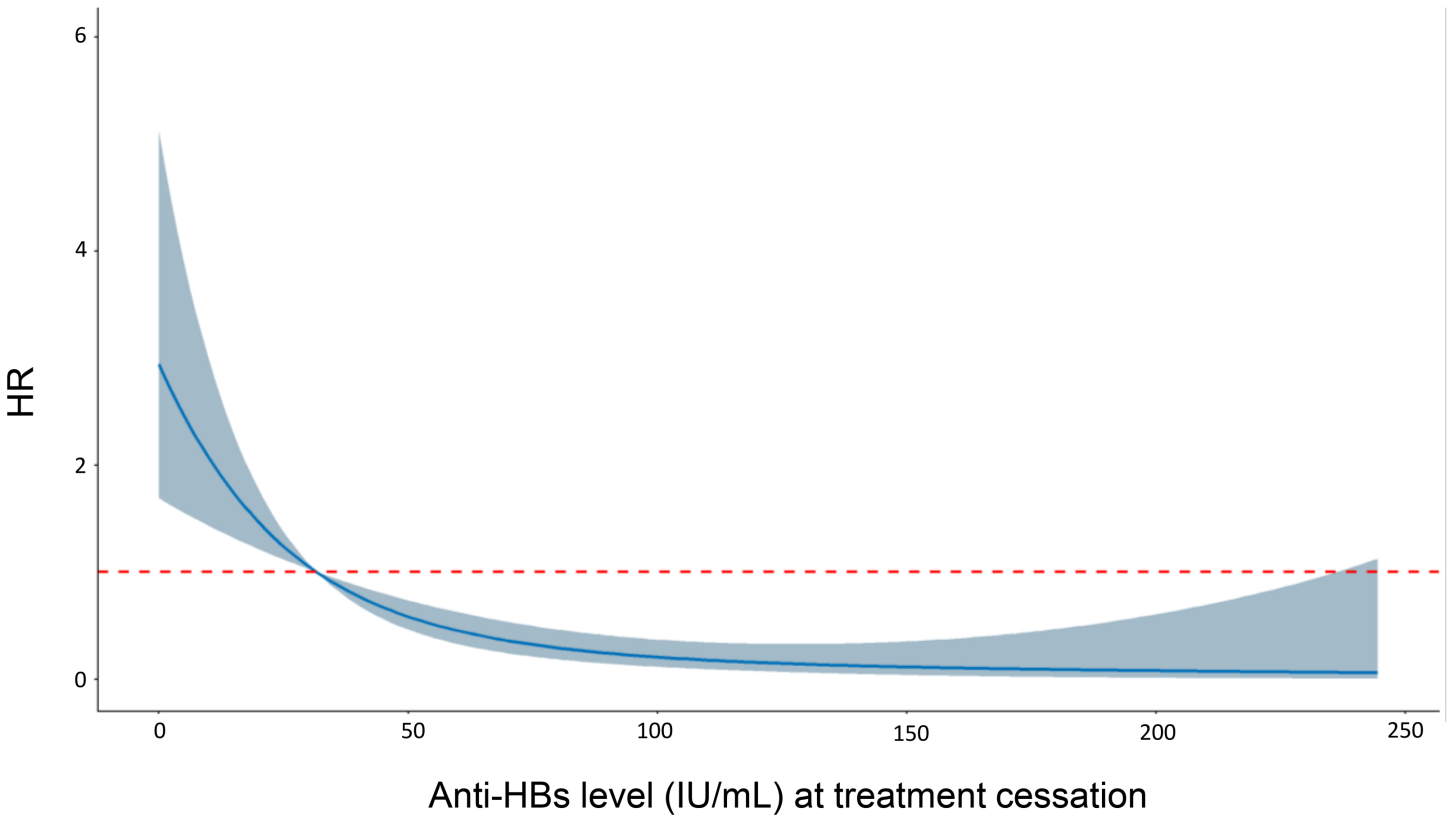
Restricted cubic spline Cox curve of anti-HBs.

Furthermore, restricted cubic spline Cox curves were plotted to examine the quantitative relationship between anti-HBs levels and the risk of HBsAg-RS after HBsAg loss (Fig. 5). The results indicated a significant linear quantitative association between anti-HBs levels and the risk of HBsAg-RS (P-Nonlinear = 0.124). This suggests that higher levels of anti-HBs at the time of treatment cessation are associated with a lower risk of HBsAg-RS.

## Discussion

It is crucial to accurately identify the population that may benefit from a limited course of Peg-IFN-*α* therapy. Our analysis, based on real-world data from our center, aligns with recent published studies (Mo et al., 2022; Xie et al., 2022) and indicates that HBsAg loss can be achieved in a short period with Peg-IFN-*α* for select patients.

However, complete eradication of hepatitis B virus is not feasible due to the persistence of HBV cccDNA and integration of HBV DNA into the host genome, leading to the possibility of HBsAg-RS after HBsAg loss (Song et al., 2021). Thus, it is crucial to identify the factors influencing HBsAg-RS after HBsAg loss to establish appropriate monitoring protocols following HBsAg clearance. In this study, we examined the factors influencing the response to Peg-IFN-*α* therapy and analyzed the factors influencing HBsAg-RS after achieving HBsAg loss through PEG-IFN-*α* therapy.

### Predictors of achieving HBsAg loss

Identifying groups that benefit from short-term Peg-IFN-*α* treatment response can have positive clinical implications, such as improving treatment adherence, reducing costs, and enhancing treatment accessibility. Our analysis highlights that the baseline HBsAg level is the most significant predictor of treatment response in the short term. Multiple regression analysis results indicate that nucleoside treatment, lower baseline HBsAg and HBV DNA levels, higher anti-HBc levels, HBeAg negativity, and greater HBsAg decline early in the treatment period are key predictors of achieving a response within a shorter treatment duration. Nucleoside-treated patients with lower HBsAg levels have a higher likelihood of response within a shorter treatment period, while it is more challenging for patients with HBeAg positivity and high HBV DNA levels at baseline to achieve HBsAg loss within a 24-week treatment course.

Recent studies have suggested that quantitative levels of anti-HBc at baseline may play a role in predicting response to interferon therapy (Brakenhoff et al., 2022; Van Campenhout et al., 2016), and combining HBsAg levels may further enhance predictive efficacy (Wang et al., 2022). We observed that higher quantitative anti-HBc levels at baseline were predictive of response over a shorter treatment period, although these levels did not remain statistically significant in further multivariate regression analyses. This may be attributed to the sample size, inter-subject variability, and differences in quantitative anti-HBc testing methods.

In the analysis of the standard treatment course (48 weeks Peg-IFN-*α* treatment), nucleoside treatment, baseline HBsAg level, and the magnitude of HBsAg decline in the early stages of treatment (at 12 or 24 weeks) were the most important factors influencing treatment response. Patients with lower baseline HBsAg levels had a higher probability of achieving HBsAg loss. Univariate analysis also revealed that the change in AST from baseline at 12 weeks, anti-HBs levels at 12 and 24 weeks, and the change in anti-HBs from baseline were predictive of eventual treatment response. However, in multivariate modeling, nucleoside treatment, baseline HBsAg level, and the magnitude of HBsAg decline early in treatment remained the most significant influences on response. These findings align with previous research (Boglione et al., 2016; Wu et al., 2020).

The treatment-responder group in the standard treatment regimen (48 weeks Peg-IFN-*α* treatment) exhibited a slight increase in anti-HBs levels early in the treatment course, whereas no such trend was observed in the non-responder group. Chronic hepatitis B infection is characterized by the failure to establish an adequate and coordinated adaptive immune response against HBV (Maini & Burton, 2019). The role of the humoral immune response in chronic hepatitis B has often been overlooked (Vanwolleghem et al., 2022). However, highly sensitive immunoassays have revealed that anti-HBs is produced and present in patients with chronic hepatitis B, but its detection is hindered by high levels of circulating HBsAg, which predominantly forms immune complexes with anti-HBs (Pernice et al., 1979). The presence of anti-HBs suggests the initial restoration and development of the body’s anti-HBV immunity, which may contribute to the achievement of HBsAg loss. This perspective is supported by clinical evidence, such as a higher rate of HBsAg loss in patients with a nonclassical serologic pattern of coexisting anti-HBs and HBsAg (Kwak et al., 2019). Additionally, the observation of a peak in HBsAg-anti-HBs immune complexes that coincides with elevated liver enzymes in patients who achieve HBsAg loss after nucleoside analog (NAs) treatment further supports the potential role of anti-HBs, as a crucial component of HBV-specific humoral immunity, in the attainment of HBsAg loss (Xu et al., 2022).

### Factors influencing the occurrence of HBsAg-RS after Peg-IFN-*α* treatment

It is crucial to understand the factors that influence the occurrence of HBsAg-RS after HBsAg loss in order to develop an effective post-HBsAg loss monitoring program. We analyzed the factors influencing HBsAg-RS after Peg-IFN-*α* treatment. By constructing cumulative risk curves and conducting one-way Cox regression analysis, we identified several factors associated with a lower risk of HBsAg-RS compared to the reference group. These factors include being younger than 50 years, receiving nucleoside treatment, having higher anti-HBc levels at the baseline of treatment, undergoing treatment consolidation for more than 3 months after HBsAg loss, and having higher levels of anti-HBs at the time of cessation of Peg-IFN-*α* treatment.

Previous studies have confirmed that individuals aged over 50 years at the time of achieving HBsAg loss after nucleoside analog (NAs) treatment have a higher risk of hepatocellular carcinoma (Yuen et al., 2008). Our analysis also revealed that age over 50 years was associated with a higher likelihood of HBsAg-RS. Age is an important factor that affects prognosis and outcomes after HBsAg loss. Some studies have reported that HBV reactivation can occur in older individuals even in the absence of traditional triggers (Kamitsukasa et al., 2015). Immune senescence associated with aging may contribute to the compromised immune control of HBV. Additionally, older age may imply longer duration of infection, which can lead to more severe HBV-specific immune tolerance and exhaustion (Le Bert et al., 2020).

Consolidation therapy with interferon has been associated with better persistence after HBsAg loss (Le Bert et al., 2020). This may be attributed to lower levels of covalently closed circular DNA (cccDNA) after consolidation therapy, which has been shown to be associated with higher anti-HBs levels (Li et al., 2019). Our findings align with this idea, highlighting the potential benefits of consolidation therapy in improving persistence after HBsAg loss.

Further multifactorial Cox regression analysis revealed that higher levels of anti-HBs at the time of discontinuation of interferon (IFN) therapy and higher levels of anti-HBc at the baseline of treatment were associated with a lower risk of HBsAg-RS after treatment. We constructed a nomogram to visualize the predicted risk of HBsAg-RS based on the levels of anti-HBs at the time of discontinuation of IFN therapy and anti-HBc at the baseline of treatment.

The level of anti-HBs at the cessation of treatment was identified as the most important factor influencing the persistence of HBsAg loss, which is consistent with previous studies (Huang et al., 2022; Li et al., 2019). In particular, there was a significant linear quantitative association between anti-HBs levels and the risk of HBsAg-RS after HBsAg loss, indicating that higher anti-HBs levels corresponded to a lower risk of HBsAg-RS.

Anti-HBs plays a crucial role in preventing viral transmission through neutralization and promoting the recovery of various immune cells involved in coordinated immune responses against HBV. It may contribute to sustained HBsAg loss by preventing reinfection or multiple rounds of infection (Ma et al., 2019). The persistence of HBsAg/anti-HBs immune complexes after HBsAg loss has been observed, and it is possible that ongoing production of HBsAg from integrated HBV DNA or residual cccDNA contributes to the pro-longed presence of HBsAg/anti-HBs immune complexes. The dynamics and equilibrium between HBsAg and anti-HBs in immune complexes may be a critical factor in the as-sociation between anti-HBs and the persistence of HBsAg loss (Ali et al., 2022; Shah et al., 2020).

The quantitative level of anti-HBc at the time of treatment cessation has been suggested to be an important predictor of HBsAg-RS, and combining it with anti-HBs levels further improves its predictive efficacy (Lin et al., 2022). In contrast, our study suggests that the level of anti-HBc at baseline may be associated with the durability of HBsAg loss, with higher levels of anti-HBc at baseline potentially leading to earlier HBsAg loss and better clearance durability. These differences may be attributed to population heterogeneity, treatment regimens, and variations in quantitative anti-HBc testing methods. Large prospective cohort studies are needed to validate the association between anti-HBc levels and the risk of HBsAg-RS.

### Limitations

We acknowledge several limitations of this study. Firstly, it was a single-center, retrospective, real-world study, and the findings may need to be further validated in prospective studies conducted in different settings. Secondly, important factors that could influence response to interferon therapy, such as HBV genotype and duration of NA , were not included in the analysis. Additionally, the unit of measurement for HBsAg quantification used in the study differs from the international standardized unit, which may limit the generalizability of the findings, despite studies showing good correlation between the two measurement methods (Shaker et al., 2012).

Finally, the durability and long-term outcomes of HBsAg loss mediated by different treatment modalities require further investigation.

## Conclusions

The findings of this study have important implications for predicting the response to Peg-IFN-*α* therapy and identifying high-risk groups for HBsAg-RS after achieving HBsAg loss. Moreover, these findings can contribute to optimizing the implementation strategy of HBsAg loss and guiding the monitoring strategy and procedures following HBsAg loss. By considering the extent of HBsAg decline at baseline and early in treatment, as well as the levels of anti-HBc and anti-HBs at baseline, healthcare professionals can better assess treatment outcomes and tailor interventions accordingly. Additionally, the significance of higher levels of anti-HBs at discontinuation and higher anti-HBc at baseline suggests the importance of monitoring and maintaining these markers for sustained HBsAg loss. Overall, these findings have practical implications for improving the management and follow-up of patients undergoing Peg-IFN-*α* therapy.

##  Supplemental Information

10.7717/peerj.20587/supp-1 Supplemental Information 1 Supplementary material

10.7717/peerj.20587/supp-2 Supplemental Information 2 24 weeks data

10.7717/peerj.20587/supp-3 Supplemental Information 3 48 weeks data

10.7717/peerj.20587/supp-4 Supplemental Information 4 Follow-up visit data

10.7717/peerj.20587/supp-5 Supplemental Information 5 24 weeks data codebook

10.7717/peerj.20587/supp-6 Supplemental Information 6 48 weeks data codebook

10.7717/peerj.20587/supp-7 Supplemental Information 7 Follow-up data codebook

## Additional Information and Declarations

### Data Availability

The following information was supplied regarding data availability: The raw data are available in the Supplemental Files.
