# Supplementary material for: Predicting loss of hepatitis B surface antigen and evaluating the durability of functional cure induced by pegylated interferon alpha: insights from a real-world study
Source: PeerJ. 2026 Jan 21;14:e20587. doi: 10.7717/peerj.20587 (PMC12831512; doi:10.7717/peerj.20587)
Supplement: Supplemental Information 1 [file peerj-14-20587-s001.docx]

Supplementary Material

**Predicting loss of hepatitis B surface antigen and evaluating the durability of functional cure induced by pegylated interferon alpha: insights from a real-world study**

***Content***

**Supplementary Results**

**Supplementary Table1.** Univariate logistic analysis affecting treatment response at 24 weeks.

**Supplementary Table 2.** Multifactorial stepwise logistic analysis affecting treatment response at 24 weeks.

**Supplementary Table 3.** Baseline information on subjects grouped according to 48-week treatment response or not.

**Supplementary Table 4.** Univariate logistic analysis affecting treatment response at 48 weeks.

**Supplementary Table 5.** Multifactorial stepwise logistic analysis affecting response to treatment at 48 weeks.

**Supplementary Table 6**. Baseline data table for subjects with or without HBsAg serologic reversal after Peg-IFN-α induced HBsAg loss.

**Supplementary Figure 1:** Area under ROC curve of different models.

**Supplementary Figure** 2: Scatter plots stratified by 24-week treatment response.

**Supplementary Figure 3:** Scatter plots stratified by 48-week treatment response.

***Supplementary Results***

A total of 265 subjects were included in the predictive analysis of treatment response for the 48-week regimen (Figure 1). Among them, 208 achieved HBsAg loss within the 48-week period, including 146 males and 62 females, with an average age of 38.9 years. Detailed baseline information of the subjects can be found in Supplementary Table 3. Comparing subjects who achieved HBsAg loss at 48 weeks to those who did not, the former had lower baseline HBsAg and HBV DNA levels, greater reductions in HBsAg and HBV DNA at 12 and 24 weeks, and a greater increase in anti-HBs levels at 24 weeks of treatment. Both groups showed some increase in ALT and AST levels and a decrease in total protein and total bilirubin levels at 12 and 24 weeks of treatment. The results of the univariate logistic analysis revealed that HBeAg positivity, baseline HBsAg and HBV DNA levels, HBsAg levels at the start 12 and 24 weeks of treatment, HBV DNA levels at 12 weeks of treatment, HBV DNA levels at 24 weeks of treatment , anti-HBs levels at 12 and 24 weeks of treatment, the change in HBsAg at 12 weeks compared to baseline, the change in HBV DNA at 12 weeks compared to baseline , the change in anti-HBs at 24 weeks compared to baseline and the change in AST at 12 weeks compared to baseline were significant predictors of response at the 48-week treatment mark (Supplementary Table 4 and Supplementary Table 5).

The area under the curve (AUC) of the logistic regression model was calculated, and the ROC curve was plotted to assess the predictive value of the metrics associated with interferon response (Supplementary Figure 1). Baseline HBsAg levels combined with nucleoside treatment predicted response at 48 weeks of treatment. The combination of baseline HBsAg levels and the decrease in HBsAg at 12 or 24 weeks of treatment further im-proved predictive efficacy (AUC: 0.902; 95% CI: 0.859-0.946).

**Supplementary Table 1**. Univariate Logistic Analysis Affecting Treatment Response at 24 Weeks

|  | Odds Ratio (95% CI) | *P* value |
| --- | --- | --- |
| Age，years | 1.254(0.818-1.922) | 0.300 |
| Gender | 0.890(0.560-1.414) | 0.890 |
| NAs | 1.538(0.992-2.385) | 0.054 |
| HBeAg (+) | 0.052(0.007-0.388) | 0.004* |
| NAFLD | 0.781(0.475-1.285) | 0.330 |
| Splenomegaly | 0.672(0.275-1.643) | 0.384 |
| Cirrhosis | 0.654(0.130-3.296) | 0.607 |
| Baselines | | |
| HBsAg (log_10_ ng/mL) | 0.400 (0.317-0.506) | 0.000* |
| Anti-HBs (mIU/mL) | 1.005 (0.992- 1.018) | 0.476 |
| Anti-HBe (log_10_ NcU/mL) | 1.218(0.964-1.538) | 0.098 |
| Anti-HBc (log_10_ NcU/mL) | 2.410(1.079-5.385) | 0.032* |
| DNA (log_10_ IU/mL) | 0.844(0.738-0.966) | 0.014* |
| AST (U/L) | 0.990(0.974-1.008) | 0.270 |
| ALT (U/L) | 0.995(0.985-1.004) | 0.280 |
| γ-GT (U/L) | 0.996(0.985-1.006) | 0.407 |
| TBIL (umol /L) | 1.014(0.987-1.042) | 0.308 |
| IBIL (umol /L) | 1.009(0.960-1.061) | 0.728 |
| TP(g/L) | 0.992(0.934-1.054) | 0.795 |
| Alb(g/L) | 0.983(0.922-1.047) | 0.591 |
| 12 Week | | |
| HBsAg (log_10_ ng/mL) | 0.195(0.130-0.292) | 0.000* |
| Anti-HBs (mIU/mL) | 1.008(0.997-1.020) | 0.144 |
| Anti-HBe (log_10_ NcU/mL) | 1.078(0.815-1.427) | 0.598 |
| Anti-HBc (log_10_ NcU/mL) | 1.203(0.609-2.377) | 0.594 |
| DNA (log_10_ IU/mL) | 0.421(0.222-0.799) | 0.008* |
| AST (U/L) | 1.000(0.995-1.006) | 0.854 |
| ALT (U/L) | 0.998(0.993-1.003) | 0.364 |
| γ-GT (U/L) | 1.002(0.999-1.004) | 0.286 |
| TBIL (umol /L) | 0.969(0.917-1.024) | 0.260 |
| IBIL (umol /L) | 0.954(0.877-1.037) | 0.270 |
| TP(g/L) | 1.004(0.946-1.066) | 0.895 |
| Alb(g/L) | 0.944(0.879-1.013) | 0.112 |
| Δ12 Week | | |
| ΔHBsAg | 3.107(2.297-4.203) | 0.000* |
| Δ Anti-HBs | 1.005(0.995-1.016) | 0.332 |
| ΔAnti-HBc | 0.745(0.375-1.480) | 0.401 |
| ΔAST | 1.002(0.997-1.007) | 0.427 |
| ΔALT | 1.001(0.997-1.005) | 0.565 |
| Δγ-GT | 1.002(0.999-1.006) | 0.163 |
| ΔTBIL | 0.979(0.944-1.014) | 0.239 |

**P* < 0.05 indicates statistical significance.

**Supplementary Table 2.** Multifactorial stepwise logistic analysis affecting treatment response at 24 weeks

|  | **Odds Ratio (95% CI)** | **P value** |
| --- | --- | --- |
| Model 1: Inclusion of only treatment baseline period variables | | |
| NAs | 1.879(1.109-3.181) | 0.019* |
| HBeAg (+) | 0.071(0.009-0.562) | 0.012* |
| HBsAg (log10 ng/mL) | 0.388(0.299-0.503) | 0.000* |
| DNA (log10 IU/mL) | 0.840(0.711-0.993) | 0.041* |
| Model 2: Inclusion of variables at treatment baseline and at 12 weeks of treatment | | |
| HBeAg (+) | 0.112(0.013-1.013) | 0.051 |
| HBsAg (log10 ng/mL) | 0.210(0.133-0.332) | 0.000* |
| DNA (log10 IU/mL) | 0.770(0.615-0.965) | 0.023* |
| ΔHBsAg (log10 ng/mL) | 5.912(3.578-9.769) | 0.000* |

*P < 0.05 indicates statistical significance.

**Supplementary Table 3.** Baseline information on subjects grouped according to 48-week treatment response or not.

|  | All | 48W no response | 48W response | *P* value |
| --- | --- | --- | --- | --- |
| N | 265 | 57 | 208 | - |
| Gender，female(%) | 75 (28.4%) | 13 (22.8%) | 62 (30.0%) | 0.372 |
| Age，years(mean±SD) | 38.63±8.09 | 37.47 (7.47%) | 38.95 (8.24%) | 0.223 |
| NAs (%) | 151 (60.2%) | 28 (50.9%) | 123 (62.8%) | 0.153 |
| HBeAg (+) (%) | 11 (4.3%) | 7 (12.7%) | 4 (2.0%) | 0.002* |
| NAFLD (%) | 81 (36.8%) | 20 (44.4%) | 61 (34.9%) | 0.310 |
| Cirrhosis (%) | 4 (1.8%) | 2 (4.3%) | 2 (1.1%) | 0.403 |
| Splenomegaly (%) | 13 (5.9%) | 5 (10.9%) | 8 (4.5%) | 0.203 |
| Baseline treatment |  |  |  |  |
| HBsAg (log_10_ ng/mL) | 1.99±1.11 | 2.81±0.90 | 1.77±1.06 | <0.001* |
| Anti-HBs (mIU/mL) | 2.51±19.25 | 0.62±1.35 | 3.03±21.69 | 0.407 |
| Anti-HBe (log_10_ NcU/mL) | 1.41±0.97 | 1.20±1.12 | 1.47±0.92 | 0.065 |
| Anti-HBc (log_10_ NcU/mL) | 2.06±0.31 | 2.01±0.30 | 2.07±0.31 | 0.206 |
| DNA (log_10_ IU/mL) | 1.03±1.58 | 1.44±1.92 | 0.92±1.46 | 0.028* |
| AST (U/L) | 25.34±12.15 | 27.49±16.42 | 24.66±10.43 | 0.165 |
| ALT (U/L) | 29.31±24.18 | 32.38±28.86 | 28.34±22.53 | 0.318 |
| γ-GT (U/L) | 26.16±18.71 | 26.49±17.90 | 26.05±19.02 | 0.890 |
| TBIL (umol /L) | 13.50±9.78 | 12.08±4.84 | 13.95±10.87 | 0.255 |
| TP(g/L) | 77.36±3.95 | 77.40±3.39 | 77.35±4.12 | 0.941 |
| 12 weeks of treatment |  |  |  |  |
| HBsAg (log_10_ ng/mL) | 0.80±1.45 | 2.28±1.27 | 0.34±1.18 | <0.001* |
| Anti-HBs (mIU/mL) | 6.51±30.50 | 0.78±1.73 | 8.23±34.60 | 0.151 |
| Anti-HBe (log_10_ NcU/mL) | 1.40±0.87 | 1.18±1.02 | 1.46±0.81 | 0.055 |
| Anti-HBc (log_10_ NcU/mL) | 2.06±0.36 | 2.03±0.41 | 2.07±0.34 | 0.542 |
| DNA (log_10_ IU/mL) | 0.14±0.58 | 0.32±0.90 | 0.09±0.44 | 0.006* |
| AST (U/L) | 66.99±48.76 | 56.10±39.73 | 70.39±50.91 | 0.097 |
| ALT (U/L) | 70.10±60.77 | 65.18±57.82 | 71.63±61.78 | 0.550 |
| γ -GT (U/L) | 81.51±77.12 | 70.45±52.98 | 84.97±83.12 | 0.288 |
| TBIL (umol /L) | 11.44±4.46 | 12.18±5.65 | 11.20±4.01 | 0.218 |
| TP (log_10_ g/L) | 74.36±4.22 | 74.85±3.36 | 74.21±4.45 | 0.392 |
| 24 weeks of treatment |  |  |  |  |
| HBsAg (ng/mL) | -0.20±1.41 | 1.23±1.23 | -0.73±1.06 | <0.001* |
| Anti-HBs (mIU/mL) | 12.85±27.63 | 1.32±2.48 | 17.02±31.20 | 0.001* |
| Anti-HBe (log_10_ NcU/mL) | 1.44±0.90 | 1.39±1.02 | 1.45±0.86 | 0.688 |
| Anti-HBc (log_10_ NcU/mL) | 2.00±0.39 | 1.95±0.44 | 2.02±0.36 | 0.243 |
| DNA (log_10_ IU/mL) | 0.08±0.43 | 0.19±0.72 | 0.05±0.30 | 0.024* |
| AST (U/L) | 56.51±40.88 | 54.32±45.58 | 57.38±39.03 | 0.665 |
| ALT (U/L) | 52.33±51.20 | 62.57±60.92 | 48.25±46.43 | 0.105 |
| γ-GT (U/L) | 83.19±97.43 | 72.89±55.89 | 87.30±109.63 | 0.393 |
| TBIL (umol /L) | 9.99±3.95 | 10.00±4.25 | 9.99±3.84 | 0.986 |
| TP (g/L) | 74.76±8.53 | 74.79±10.13 | 74.75±7.85 | 0.976 |
| Change from baseline at 12 weeks |  |  |  |  |
| ΔHBsAg (log_10_ ng/mL) | 1.24±1.03 | 0.63±0.75 | 1.43±1.03 | <0.001* |
| ΔAnti-HBs (mIU/mL) | 3.51±30.35 | 0.16±1.57 | 4.52±34.54 | 0.405 |
| ΔAnti-HBe (log_10_ NcU/mL) | -0.02±0.37 | 0.06±0.50 | -0.05±0.32 | 0.080 |
| ΔAnti-HBc (log_10_ NcU/mL) | -0.02±0.36 | 0.02±0.43 | 2.66±1.16 | 0.391 |
| ΔAST (U/L) | 41.96±50.36 | 26.39±31.79 | 47.35±54.46 | 0.031* |
| ΔALT (U/L) | 43.50±60.22 | 31.60±49.78 | 47.62±63.12 | 0.170 |
| Δγ-GT (U/L) | 56.88±75.08 | 41.75±39.32 | 62.17±83.59 | 0.161 |
| ΔTBIL (umol /L) | -2.08±9.46 | -1.59±4.37 | -2.25±10.70 | 0.718 |
| ΔTP (g/L) | -3.14±4.25 | -2.70±3.35 | -3.30±4.53 | 0.474 |
| Change from baseline at 24 weeks |  |  |  |  |
| ΔHBsAg (log_10_ ng/mL) | 2.39±1.22 | 1.62±1.04 | -0.03±0.33 | <0.001* |
| ΔAnti-HBs (mIU/mL) | 11.91±27.48 | 0.73±2.63 | 15.92±31.05 | 0.001* |
| ΔAnti-HBe (log10 NcU/mL) | 0.04±0.59 | 0.04±0.65 | 0.03±0.57 | 0.938 |
| ΔAnti-HBc (log10 NcU/mL) | -0.04±0.39 | -0.06±0.43 | -0.04±0.37 | 0.790 |
| ΔAST (U/L) | 31.15±35.17 | 29.61±40.30 | 31.87±32.71 | 0.735 |
| ΔALT (U/L) | 25.52±41.23 | 33.66±55.94 | 21.68±31.75 | 0.126 |
| Δγ-GT (U/L) | 56.70±84.18 | 50.12±48.74 | 59.80±96.58 | 0.546 |
| ΔTBIL (umol /L) | -3.90±10.63 | -2.73±4.18 | -4.45±12.56 | 0.395 |
| ΔTP (g/L) | -2.37±6.70 | -3.10±10.09 | -2.03±4.30 | 0.398 |

*P < 0.05 indicates statistical significance.

**Supplementary Table 4.** Univariate Logistic Analysis Affecting Treatment Response at 48 Weeks.

|  | OR (95% CI) | *P* value |
| --- | --- | --- |
| Age，years | 0.691(0.348-1.373) | 0.291 |
| Gender | 1.024(0.986-1.063) | 0.223 |
| NAs | 1.625(0.889-2.969) | 0.115 |
| HBeAg (+) | 0.139(0.039-0.495) | 0.002* |
| NAFLD | 0.669(0.344-1.301) | 0.236 |
| Cirrhosis | 0.253(0.035-1.846) | 0.175 |
| Splenomegaly | 0.390(0.121-1.256) | 0.115 |
| Baseline treatment | | |
| HBsAg (log10 ng/mL) | 0.343(0.236-0.498) | 0.000* |
| Anti-HBs (mIU/mL) | 1.069(0.905-1.262) | 0.433 |
| Anti-HBe (log_10_ NcU/mL) | 1.315(0.980-1.766) | 0.068 |
| Anti-HBc (log_10_ NcU/mL) | 1.794(0.721-4.466) | 0.209 |
| DNA (log_10_ IU/mL) | 0.825(0.694-0.982) | 0.030* |
| AST (U/L) | 0.983(0.960-1.008) | 0.177 |
| ALT (U/L) | 0.994(0.981-1.006) | 0.322 |
| γ-GT (U/L) | 0.999(0.982-1.016) | 0.889 |
| TBIL (umol /L) | 1.034(0.976-1.094) | 0.257 |
| TP(g/L) | 0.997(0.917-1.083) | 0.940 |
| Treatment for 12 weeks | | |
| HBsAg (log_10_ ng/mL) | 0.330(0.237-0.459) | 0.000* |
| Anti-HBs (mIU/mL) | 1.234(1.022-1.489) | 0.028* |
| Anti-HBe (log_10_ NcU/mL) | 1.445(0.988-2.114) | 0.058 |
| Anti-HBc (log_10_ NcU/mL) | 1.327(0.536-3.285) | 0.541 |
| DNA (log_10_ IU/mL) | 0.537(0.372-0.883) | 0.012* |
| AST (U/L) | 1.008(0.998-1.018) | 0.106 |
| ALT (U/L) | 1.002(0.996-1.008) | 0.549 |
| γ-GT (U/L) | 1.003(0.997-1.009) | 0.293 |
| TBIL (umol /L) | 0.956(0.889-1.028) | 0.227 |
| TP(g/L) | 0.964(0.887-1.048) | 0.390 |
| Treatment for 24 weeks | | |
| HBsAg (log_10_ ng/mL) | 0.207(0.122-0.352) | 0.000* |
| Anti-HBs (mIU/mL) | 1.232(1.074-1.413) | 0.003* |
| Anti-HBe (log_10_ NcU/mL) | 1.078(0.749-1.552) | 0.686 |
| Anti-HBc (log_10_ NcU/mL) | 1.626(0.717-3.687) | 0.244 |
| AST (U/L) | 1.002(0.993-1.011) | 0.664 |
| ALT (U/L) | 0.995(0.989-1.001) | 0.125 |
| γ-GT (U/L) | 1.002(0.997-1.007) | 0.404 |
| TBIL (umol /L) | 0.999(0.917-1.089) | 0.986 |
| TP(g/L) | 0.999(0.960-1.040) | 0.976 |
| Change from baseline at 12 weeks | | |
| ΔHBsAg (log_10_ ng/mL) | 2.904(1.770-4.764) | 0.000* |
| ΔAnti-HBs (mIU/mL) | 1.006(0.992-1.020) | 0.406 |
| ΔAnti-HBe (log_10_ NcU/mL) | 0.483(0.205-1.134) | 0.095 |
| ΔAnti-HBc (log_10_ NcU/mL) | 0.654(0.249-1.720) | 0.389 |
| ΔAST (U/L) | 1.012(1.001-1.024) | 0.038* |
| ΔALT (U/L) | 1.005(0.998-1.013) | 0.172 |
| Δγ-GT (U/L) | 1.005(0.998-1.012) | 0.174 |
| ΔTBIL (umol /L) | 0.991(0.943-1.042) | 0.721 |
| ΔTP (g/L) | 0.967(0.884-1.058) | 0.471 |
| Change from baseline at 24 weeks | | |
| ΔHBsAg (log_10_ ng/mL) | 2.283(1.618-3.221) | 0.000* |
| ΔAnti-HBs (mIU/mL) | 1.160(1.043-1.290) | 0.006* |
| ΔAnti-HBe (log_10_ NcU/mL) | 0.977(0.554-1.724) | 0.937 |
| ΔAnti-HBc (log_10_ NcU/mL) | 1.127(0.470-2.703) | 0.789 |
| ΔAST (U/L) | 1.002(0.991-1.013) | 0.733 |
| ΔALT (U/L) | 0.993(0.984-1.002) | 0.138 |
| Δγ-GT (U/L) | 1.002(0.996-1.007) | 0.552 |
| ΔTBIL (umol /L) | 0.978(0.928-1.032) | 0.418 |
| ΔTP(g/L) | 1.023(0.968-1.081) | 0.415 |

*P < 0.05 indicates statistical significance.

**Supplementary Table 5**. Multifactorial stepwise logistic analysis affecting response to treatment at 48 weeks

|  | **OR (95% CI)** | **P value** |
| --- | --- | --- |
| Model 1: Inclusion of only treatment baseline period variables | | |
| NAs | 2.860(1.308-6.254) | 0.008* |
| Baseline HBsAg (log10 ng/mL) | 0.351(0.230-0.536) | 0.000* |
| Model 2: Inclusion of variables at treatment baseline and at 12 weeks of treatment | | |
| Baseline HBsAg (log10 ng/mL) | 0.254(0.126-0.512) | 0.000* |
| ΔHBsAg (log10 ng/mL) | 3.582(1.788-7.179) | 0.000* |
| Model 3: Inclusion of variables at treatment baseline and at 24 weeks of treatment | | |
| Baseline HBsAg (log10 ng/mL) | 0.177(0.077-0.405) | 0.000* |
| ΔHBsAg (log10 ng/mL) | 5.654(2.488-12.848) | 0.000* |

*P < 0.05 indicates statistical significance.

**Supplementary Table 6.** Baseline data table for subjects with or without rehabilitation for 48 weeks

|  | All | Persistent response over 48 weeks (65 cases) | Seroconversion within 48 weeks (36 cases) | *P* value |
| --- | --- | --- | --- | --- |
| Age，years | 40.54±8.04 | 40.91±7.25 | 39.89±9.38 | 0.545 |
| Age > 50 years | 16 (15.8%) | 7 (10.8%) | 9 (25.0%) | 0.112 |
| Gender，female | 29 (28.7%) | 18 (27.7%) | 11 (30.6%) | 0.940 |
| NAs (%) | 67 (66.3%) | 50 (76.9%) | 17 (47.2%) | 0.005* |
| Baseline HBeAg positivity | 1 (1.0%) | 1 (1.5%) | 0 (0.0%) | 1.000 |
| Combined NAFLD | 33 (32.7%) | 20 (30.8%) | 13 (36.1%) | 0.744 |
| Combined cirrhosis | 2 (2.0%) | 2 (3.1%) | 0 (0.0%) | 0.751 |
| Combined splenomegaly | 4 (4.0%) | 4 (6.2%) | 0 (0.0%) | 0.324 |
| Duration of IFN consolidation >12 weeks after response | 51 (50.5%) | 39 (60.0%) | 12 (33.3%) | 0.018* |
| Hepatitis B vaccination after HBsAg clearance | 78 (77.2%) | 50 (76.9%) | 28 (77.8%) | 1.000 |
| Serological indicators at baseline treatment | | | | |
| HBsAg (log_10_ ng/mL) | 1.83±1.07 | 1.90±1.14 | 1.71±0.94 | 0.399 |
| Anti-HBs (mIU/mL) | 3.74±26.06 | 5.37±32.43 | 0.80±1.75 | 0.401 |
| Anti-HBe (log_10_ NcU/mL) | 1.57±0.96 | 1.48±0.99 | 1.73±0.88 | 0.217 |
| Anti-HBc (log_10_ NcU/mL) | 2.08±0.36 | 2.11±0.31 | 2.00±0.42 | 0.145 |
| Serological indicators at cessation of PEG-IFN-alpha therapy | | | | |
| Anti-HBs (mIU/mL) | 71.50±89.97 | 102.17±96.76 | 16.11±33.21 | <0.001* |
| Anti-HBe (log_10_ NcU/mL) | 1.57±0.76 | 1.50±0.79 | 1.70±0.68 | 0.187 |
| Anti-HBc (log_10_ NcU/mL) | 1.99±0.42 | 1.99±0.38 | 2.00±0.49 | 0.846 |

*P<0.05 indicates statistical significance.


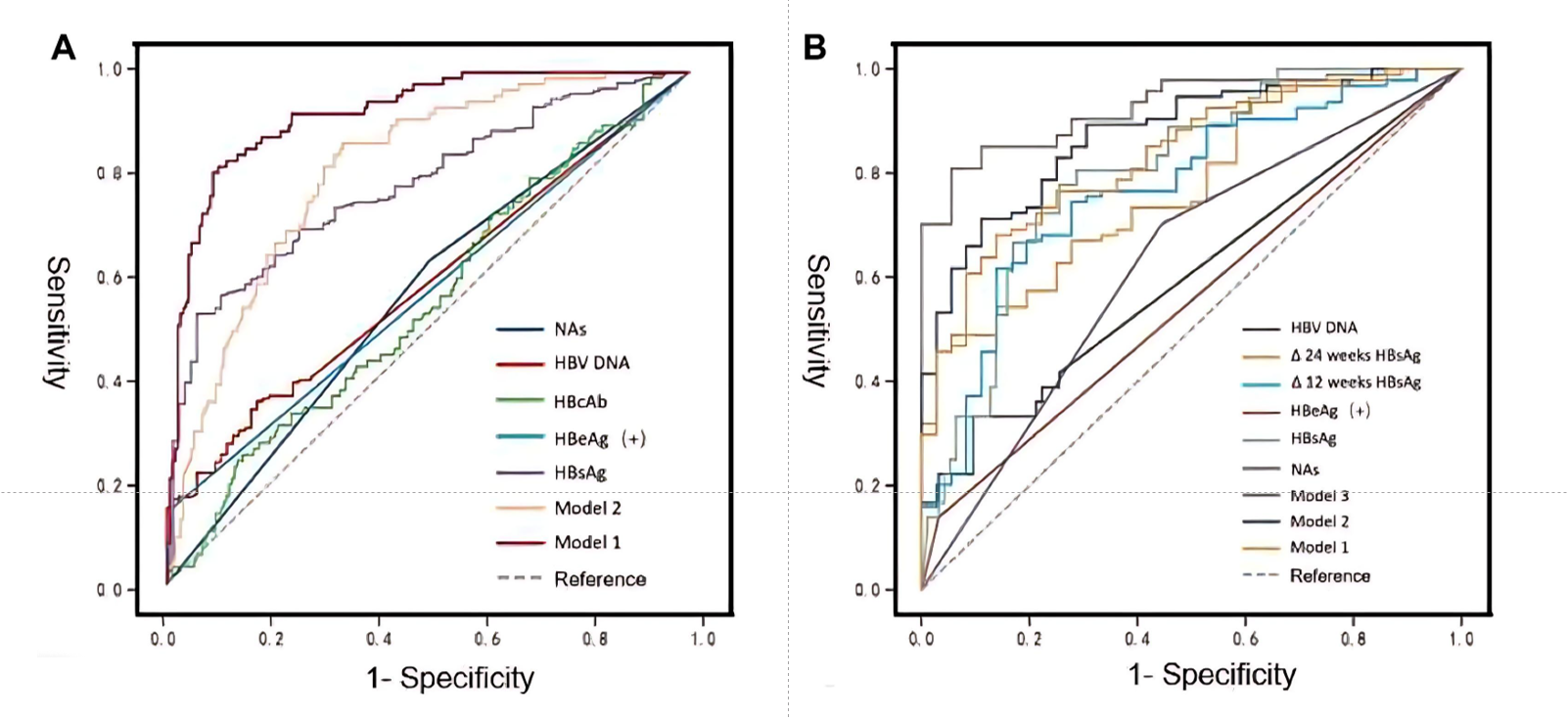
 Supplementary Figure 1: Area under ROC curve of different models. a) Model 1 consisted of treated nucleoside +HBeAg positive + baseline HBsAg+ baseline HBV DNA; Model 2 was HBeAg positive + baseline HBsAg + baseline HBV DNA+ΔHBsAg. b) Model 1 was nucleoside therapy + baseline HBsAg; Model 2 was baseline HBsAg + ΔHBsAg at 12 weeks; Model 3 was baseline HBsAg + ΔHBsAg at 24 weeks.


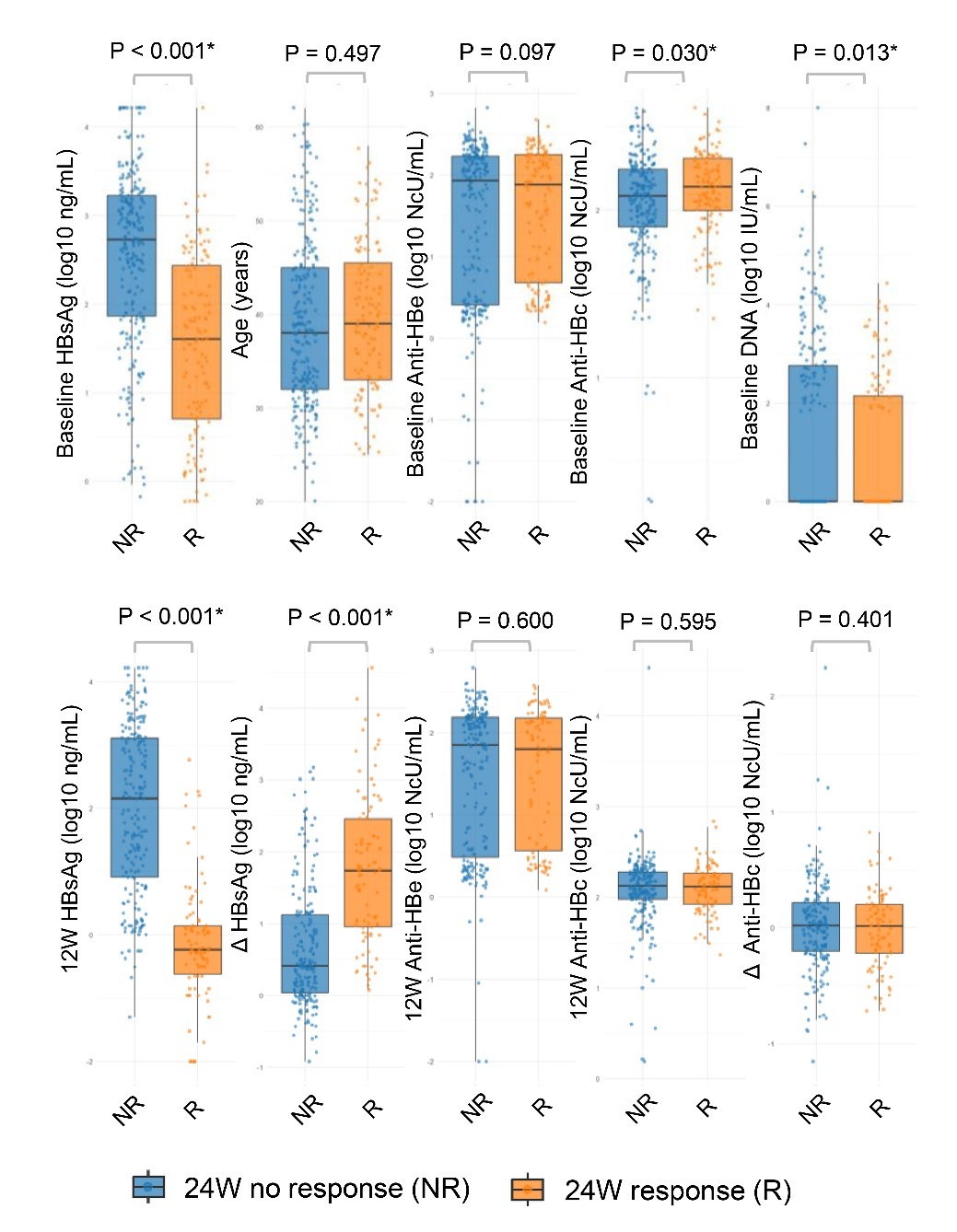


**Supplementary Figure** 2: Scatter plots stratified by 24-week treatment response.


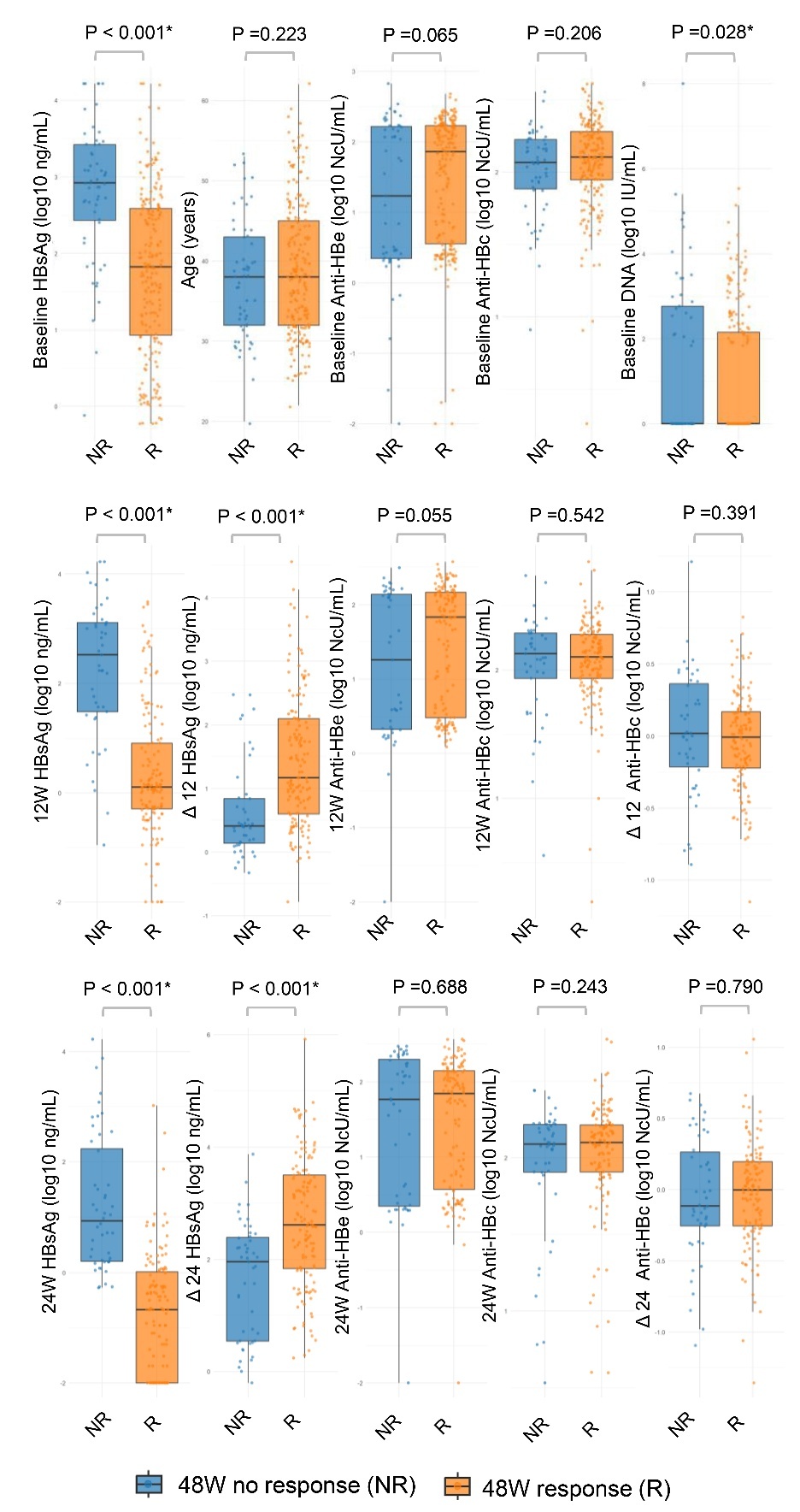


**Supplementary Figure 3:** Scatter plots stratified by 48-week treatment response.
