# Supplementary material for: Predicting loss of hepatitis B surface antigen and evaluating the durability of functional cure induced by pegylated interferon alpha: insights from a real-world study
Source: PeerJ. 2026 Jan 21;14:e20587. doi: 10.7717/peerj.20587 (PMC12831512; doi:10.7717/peerj.20587)
Supplement: Supplemental Information 5 [file peerj-14-20587-s005.docx]

| **Variable name** | **Meaning** | **Categorical variable** |
| --- | --- | --- |
| ID-24 week | Serial Number |  |
| Response | Response at 24 weeks of treatment | 1=response; 0=no response |
| SEX | Sex | 1=male; 0=female |
| AGE | Age |  |
| age>50y | Is the age greater than 50 years old? | 1=yes;0=no |
| NAs | Use Nucleos (t)ide Analogues or not | 1=yes;0=no |
| Cirrhosis | With or without liver cirrhosis | 1=yes;0=no |
| Fatty liver | With or without fatty liver | 1=yes;0=no |
| Splenomegaly | With or without enlarged spleen | 1=yes;0=no |
| HBsAg | Hepatitis B Surface Antigen (ng/mL) at baseline | |
| HBsAb | Hepatitis B Surface Antibody(mIU/mL) at baseline | |
| HBeAg | Hepatitis B e Antigen (NcU/mL) at baseline | |
| HBeAb | Hepatitis B e Antibody (NcU/mL) at baseline | |
| HBcAb | Hepatitis B Core Antibody (NcU/mL) at baseline | |
| DNA | HBV DNA (log10 IU/mL) at baseline | |
| RBC | Red Blood Cell (10 ^12 /L) at baseline | |
| PLT | Platelet (10 ^9 /L) at baseline | |
| LYM | Lymphocyte (10 ^9 /L) at baseline | |
| AST | Aspartate Aminotransferase (U/L) at baseline | |
| ALT | Alanine Aminotransferase (U/L) at baseline | |
| Y-GT | Gamma-Glutamyl Transferase (U/L) at baseline | |
| TBIL | Total Bilirubin (umol /L) at baseline | |
| IBIL | Indirect Bilirubin (umol /L) at baseline | |
| TP | Total Protein (g/L) at baseline | |
| Alb | Albumin (g/L) at baseline | |
| eGFR | Estimated Glomerular Filtration Rate (mL/min/1.73m²) at baseline | |
| UA | Uric Acid (umol /L) at baseline | |
| 12HBsAg | Hepatitis B Surface Antigen (ng/mL) at 12 weeks | |
| 12HBsAb | Hepatitis B Surface Antibody(mIU/mL) at 12 weeks | |
| 12HBeAg | Hepatitis B e Antigen (NcU/mL) at 12 weeks | |
| 12HBeAb | Hepatitis B e Antibody (NcU/mL) at 12 weeks | |
| 12HBcAb | Hepatitis B Core Antibody (NcU/mL) at 12 weeks | |
| 12DNA | HBV DNA (log10 IU/mL) at 12 weeks | |
| 12WBC | White Blood Cell (10 ^12 /L) at 12 weeks | |
| 12RBC | Red Blood Cell (10 ^12 /L) at 12 weeks | |
| 12PLT | Platelet (10 ^9 /L) at 12 weeks | |
| 12LYM | Lymphocyte (10 ^9 /L) at 12 weeks | |
| 12AST | Aspartate Aminotransferase (U/L) at 12 weeks | |
| 12ALT | Alanine Aminotransferase (U/L) at 12 weeks | |
| 12Y-GT | Gamma-Glutamyl Transferase (U/L)at 12 weeks | |
| 12TBIL | Total Bilirubin (umol /L)at 12 weeks | |
| 12IBIL | Indirect Bilirubin (umol /L) at 12 weeks | |
| 12TP | Total Protein (g/L) at 12 weeks | |
| 12Alb | Albumin (g/L) at 12 weeks | |
| 12eGFR | Estimated Glomerular Filtration Rate (mL/min/1.73m²) at 12 weeks | |
| 12UA | Uric Acid (umol /L) at 12 weeks | |
| cHBsAg | The change in HBsAg from week 12 to the baseline value | |
| cHBcAb | The change in HBcAb from week 12 to the baseline value | |
| cAST | The change in AST from week 12 to the baseline value | |
| cALT | The change in ALT from week 12 to the baseline value | |
| cY-GT | The change in Y-GT from week 12 to the baseline value | |
| Ctbil | The change in TBIL from week 12 to the baseline value | |
| L12HBsAg | Hepatitis B Surface Antigen (log 10 ng/mL) at 12 weeks | |
| L12HBeAb | Hepatitis B e Antibody (log 10 NcU/mL) at 12 weeks | |
| L12HBcAb | Hepatitis B Core Antibody (log 10NcU/mL) at 12 weeks | |
| L12HBeAg | Hepatitis B e Antigen (log 10 NcU/mL) at 12 weeks | |
