# Supplementary material for: Predicting loss of hepatitis B surface antigen and evaluating the durability of functional cure induced by pegylated interferon alpha: insights from a real-world study
Source: PeerJ. 2026 Jan 21;14:e20587. doi: 10.7717/peerj.20587 (PMC12831512; doi:10.7717/peerj.20587)
Supplement: Supplemental Information 6 [file peerj-14-20587-s006.docx]

| **Variable name** | **Meaning** | **Categorical variable** |
| --- | --- | --- |
| Response | Response at 48 weeks of treatment | 1=response; 0=no response |
| id-48w | Serial Number |  |
| AGE | Age |  |
| SEX | Sex | 1=male; 0=female |
| NAs | Use Nucleos (t)ide Analogues or not | 1=yes;0=no |
| Cirrhosis | With or without liver cirrhosis | 1=yes;0=no |
| Fatty liver | With or without fatty liver | 1=yes;0=no |
| Splenomegaly | With or without enlarged spleen | 1=yes;0=no |
| HBsAg | Hepatitis B Surface Antigen (ng/mL) at baseline |  |
| LHBsAg | Hepatitis B Surface Antigen (log 10 ng/mL) at baseline |  |
| HBsAb | Hepatitis B Surface Antibody(mIU/mL) at baseline |  |
| HBeAg | Hepatitis B e Antigen (NcU/mL) at baseline |  |
| HBeAb | Hepatitis B e Antibody (NcU/mL) at baseline |  |
| LHBeAb | Hepatitis B e Antibody (log 10 NcU/mL) at baseline |  |
| HBcAb | Hepatitis B Core Antibody (NcU/mL) at baseline |  |
| LHBcAb | Hepatitis B Core Antibody (log 10 NcU/mL) at baseline |  |
| DNA | HBV DNA (log10 IU/mL) at baseline |  |
| WBC | White Blood Cell (10 ^12 /L) at baseline |  |
| RBC | Red Blood Cell (10 ^12 /L) at baseline |  |
| PLT | Platelet (10 ^9 /L) at baseline |  |
| LYM | Lymphocyte (10 ^9 /L) at baseline |  |
| AST | Aspartate Aminotransferase (U/L) at baseline |  |
| ALT | Alanine Aminotransferase (U/L) at baseline |  |
| Y-GT | Gamma-Glutamyl Transferase (U/L) at baseline |  |
| TBIL | Total Bilirubin (umol /L) at baseline |  |
| IBIL | Indirect Bilirubin (umol /L) at baseline |  |
| TP | Total Protein (g/L) at baseline |  |
| Alb | Albumin (g/L) at baseline |  |
| eGFR | Estimated Glomerular Filtration Rate (mL/min/1.73m²) at baseline |  |
| UA | Uric Acid (umol /L) at baseline |  |
| 12HBsAg | Hepatitis B Surface Antigen (ng/mL) at 12 weeks |  |
| L12HBsAg | Hepatitis B Surface Antigen (log10 ng/mL) at 12 weeks |  |
| 12HBsAb | Hepatitis B Surface Antibody(mIU/mL) at 12 weeks |  |
| 12HBeAg | Hepatitis B e Antigen (NcU/mL) at 12 weeks |  |
| 12HBeAb | Hepatitis B e Antibody (NcU/mL) at 12 weeks |  |
| L12HBeAb | Hepatitis B e Antibody (log10 NcU/mL) at 12 weeks |  |
| 12HBcAb | Hepatitis B Core Antibody (NcU/mL) at 12 weeks |  |
| L12HBcAb | Hepatitis B Core Antibody (log10 NcU/mL) at 12 weeks |  |
| 12HBV DNA | HBV DNA (log10 IU/mL) at 12 weeks |  |
| 12WBC | White Blood Cell (10 ^12 /L) at 12 weeks |  |
| 12RBC | Red Blood Cell (10 ^12 /L) at 12 weeks |  |
| 12PLT | Platelet (10 ^9 /L) at 12 weeks |  |
| 12LYM | Lymphocyte (10 ^9 /L) at 12 weeks |  |
| 12AST | Aspartate Aminotransferase (U/L) at 12 weeks |  |
| 12ALT | Alanine Aminotransferase (U/L) at 12 weeks |  |
| 12Y-GT | Gamma-Glutamyl Transferase (U/L)at 12 weeks |  |
| 12TBIL | Total Bilirubin (umol /L)at 12 weeks |  |
| 12IBIL | Indirect Bilirubin (umol /L) at 12 weeks |  |
| 12TP | Total Protein (g/L) at 12 weeks |  |
| 12Alb | Albumin (g/L) at 12 weeks |  |
| 12eGFR | Estimated Glomerular Filtration Rate (mL/min/1.73m²) at 12 weeks |  |
| 12UA | Uric Acid (umol /L) at 12 weeks |  |
| 24HBsAg | Hepatitis B Surface Antigen (ng/mL) at 24 weeks |  |
| L24HBsAg | Hepatitis B Surface Antigen (log10 ng/mL) at 24 weeks |  |
| 24HBsAb | Hepatitis B Surface Antibody(mIU/mL) at 24 weeks |  |
| 24HBeAg | Hepatitis B e Antibody (NcU/mL) at 24 weeks |  |
| 24HBeAb | Hepatitis B e Antibody (NcU/mL) at 24 weeks |  |
| L24HBeAb | Hepatitis B e Antibody (log10 NcU/mL) at 24 weeks |  |
| 24HBcAb | Hepatitis B Core Antibody (NcU/mL) at 24 weeks |  |
| L24HBcAb | Hepatitis B Core Antibody (log10 NcU/mL) at 24 weeks |  |
| 24HBV DNA | HBV DNA (log10 IU/mL) at 24 weeks |  |
| 24WBC | White Blood Cell (10 ^12 /L) at 24 weeks |  |
| 24RBC | Red Blood Cell (10 ^12 /L) at 24 weeks |  |
| 24PLT | Platelet (10 ^9 /L) at 24 weeks |  |
| 24LYM | Lymphocyte (10 ^9 /L) at 24 weeks |  |
| 24AST | Aspartate Aminotransferase (U/L) at 24 weeks |  |
| 24ALT | Alanine Aminotransferase (U/L) at 24 weeks |  |
| 24Y-GT | Gamma-Glutamyl Transferase (U/L)at 24 weeks |  |
| 24TBIL | Total Bilirubin (umol /L)at 24 weeks |  |
| 24IBIL | Indirect Bilirubin (umol /L) at 24 weeks |  |
| 24TP | Total Protein (g/L) at 24 weeks |  |
| 24Alb | Albumin (g/L) at 24 weeks |  |
| 24eGFR | Estimated Glomerular Filtration Rate (mL/min/1.73m²) at 24 weeks |  |
| 24UA | Uric Acid (umol /L) at 24 weeks |  |
| c12sAg | The change in HBsAg from week 12 to the baseline value |  |
| c24sAg | The change in HBsAg from week 24 to the baseline value |  |
| c12HBcAb | The change in HBcAb from week 12 to the baseline value |  |
| c24HBcAb | The change in HBcAb from week 24 to the baseline value |  |
| c12HBsAb | The change in HBsAb from week 12 to the baseline value |  |
| c12HBeAb | The change in HBeAb from week 12 to the baseline value |  |
| c12AST | The change in AST from week 12 to the baseline value |  |
| c12ALT | The change in ALT from week 12 to the baseline value |  |
| c12Y-GT | The change in Y-GT from week 12 to the baseline value |  |
| c12TBIL | The change in TBIL from week 12 to the baseline value |  |
| c12TP | The change in TP from week 12 to the baseline value |  |
| C12alb | The change in ALB from week 12 to the baseline value |  |
| c24HBsAb | The change in HBsAg from week 24 to the baseline value |  |
| c24HBeAb | The change in HBeAb from week 24 to the baseline value |  |
| c24HBcAB | The change in HBcAb from week 24 to the baseline value |  |
| c24AST | The change in AST from week 24 to the baseline value |  |
| c24ALT | The change in ALT from week 24 to the baseline value |  |
| c24GT | The change in GT from week 24 to the baseline value |  |
| c24TBIL | The change in TBIL from week 24 to the baseline value |  |
| c24TP | The change in TP from week 24 to the baseline value |  |
| c24ALB | The change in ALB from week 24 to the baseline value |  |
