# Supplementary material for: Predicting loss of hepatitis B surface antigen and evaluating the durability of functional cure induced by pegylated interferon alpha: insights from a real-world study
Source: PeerJ. 2026 Jan 21;14:e20587. doi: 10.7717/peerj.20587 (PMC12831512; doi:10.7717/peerj.20587)
Supplement: Supplemental Information 7 [file peerj-14-20587-s007.docx]

| **Variable name** | **Meaning** | **Categorical variable** |
| --- | --- | --- |
| Consolidate | Whether to consolidate with IFN for more than 3 months after response | 0= no； 1=yes |
| id | Serial Number |  |
| vaccine | Receive vaccination after response | 0= no； 1=yes |
| AGE | Age |  |
| SEX | Sex | 1=male; 0=female |
| NAs | Use Nucleos (t)ide Analogues or not | 1=yes;0=no |
| Cirrhosis | With or without liver cirrhosis | 1=yes;0=no |
| Fatty liver | With or without fatty liver | 1=yes;0=no |
| Splenomegaly | With or without enlarged spleen | 1=yes;0=no |
| HBsAg | Hepatitis B Surface Antigen (ng/mL) at baseline |  |
| HBsAb | Hepatitis B Surface Antibody(mIU/mL) at baseline |  |
| HBeAg | Hepatitis B e Antigen (NcU/mL) at baseline |  |
| HBeAb | Hepatitis B e Antibody (NcU/mL) at baseline |  |
| HBcAb | Hepatitis B Core Antibody (NcU/mL) at baseline |  |
| DNA | HBV DNA (log10 IU/mL) at baseline |  |
| AST | Aspartate Aminotransferase (U/L) at baseline |  |
| ALT | Alanine Aminotransferase (U/L) at baseline |  |
| Y-GT | Gamma-Glutamyl Transferase (U/L) at baseline |  |
| TBIL | Total Bilirubin (umol /L) at baseline |  |
| IBIL | Indirect Bilirubin (umol /L) at baseline |  |
| TP | Total Protein (g/L) at baseline |  |
| Alb | Albumin (g/L) at baseline |  |
| 12HBsAg | Hepatitis B Surface Antigen (ng/mL) at 12 weeks |  |
| 12HBsAb | Hepatitis B Surface Antibody(mIU/mL) at 12 weeks |  |
| 12HBeAg | Hepatitis B e Antigen (NcU/mL) at 12 weeks |  |
| 12HBeAb | Hepatitis B e Antibody (NcU/mL) at 12 weeks |  |
| 12HBcAb | Hepatitis B Core Antibody (NcU/mL) at 12 weeks |  |
| 12HBV DNA | HBV DNA (log10 IU/mL) at 12 weeks |  |
| 12WBC | White Blood Cell (10 ^12 /L) at 12 weeks |  |
| 12RBC | Red Blood Cell (10 ^12 /L) at 12 weeks |  |
| 12PLT | Platelet (10 ^9 /L) at 12 weeks |  |
| 12LYM | Lymphocyte (10 ^9 /L) at 12 weeks |  |
| 12AST | Aspartate Aminotransferase (U/L) at 12 weeks |  |
| 12ALT | Alanine Aminotransferase (U/L) at 12 weeks |  |
| 12Y-GT | Gamma-Glutamyl Transferase (U/L)at 12 weeks |  |
| 12TBIL | Total Bilirubin (umol /L)at 12 weeks |  |
| 12IBIL | Indirect Bilirubin (umol /L) at 12 weeks |  |
| 12TP | Total Protein (g/L) at 12 weeks |  |
| 12Alb | Albumin (g/L) at 12 weeks |  |
| 24HBsAg | Hepatitis B Surface Antigen (ng/mL) at 24 weeks |  |
| 24HBsAb | Hepatitis B Surface Antibody(mIU/mL) at 24 weeks |  |
| 24HBeAg | Hepatitis B e Antigen (NcU/mL) at 24 weeks |  |
| 24HBeAb | Hepatitis B e Antibody (NcU/mL) at 24 weeks |  |
| 24HBcAb | Hepatitis B Core Antibody (NcU/mL) at 24 weeks |  |
| 24HBV DNA | HBV DNA (log10 IU/mL) at 24 weeks |  |
| 24WBC | White Blood Cell (10 ^12 /L) at 24 weeks |  |
| 24RBC | Red Blood Cell (10 ^12 /L) at 24 weeks |  |
| 24PLT | Platelet (10 ^9 /L) at 24 weeks |  |
| 24LYM | Lymphocyte (10 ^9 /L) at 24 weeks |  |
| 24AST | Aspartate Aminotransferase (U/L) at 24 weeks |  |
| 24ALT | Alanine Aminotransferase (U/L) at 24 weeks |  |
| 24Y-GT | Gamma-Glutamyl Transferase (U/L)at 24 weeks |  |
| 24TBIL | Total Bilirubin (umol /L)at 24 weeks |  |
| 24IBIL | Indirect Bilirubin (umol /L) at 24 weeks |  |
| 24TP | Total Protein (g/L) at 24 weeks |  |
| 24Alb | Albumin (g/L) at 24 weeks |  |
| c12sAg | The change in HBsAg from week 12 to the baseline value |  |
| c24sAg | The change in HBsAg from week 24 to the baseline value |  |
| c12HBcAb | The change in HBcAb from week 12 to the baseline value |  |
| c24HBcAb | The change in HBcAb from week 24 to the baseline value |  |
| zyHBsAg | Hepatitis B Surface Antigen (ng/mL) at the time of response |  |
| zyHBsAb | Hepatitis B Surface Antibody(mIU/mL) at the time of response |  |
| zyHBeAg | Hepatitis B e Antigen (NcU/mL) at the time of response |  |
| zyHBeAb | Hepatitis B e Antibody (NcU/mL) at the time of response |  |
| zyHBcAb | Hepatitis B Core Antibody (NcU/mL) at the time of response |  |
| zyHBV DNA | HBV DNA (log10 IU/mL) at the time of response |  |
| zyWBC | White Blood Cell (10 ^12 /L) at the time of response |  |
| zyRBC | Red Blood Cell (10 ^12 /L) at the time of response |  |
| zyPLT | Platelet (10 ^9 /L) at the time of response |  |
| zyLYM | Lymphocyte (10 ^9 /L) at the time of response |  |
| zyAST | Aspartate Aminotransferase (U/L)at the time of response |  |
| zyALT | Alanine Aminotransferase (U/L) at the time of response |  |
| zyY-GT | Gamma-Glutamyl Transferase (U/L)atat the time of response |  |
| zyTBIL | Total Bilirubin (umol /L)at the time of response |  |
| zyIBIL | Indirect Bilirubin (umol /L) at the time of response |  |
| zyTP | Total Protein (g/L) at the time of response |  |
| zyAlb | Albumin (g/L) at the time of response |  |
| sfHBsAg | Hepatitis B Surface Antigen (ng/mL) at Follow-up Visit |  |
| sfHBsAb | Hepatitis B Surface Antibody(mIU/mL) at Follow-up Visit |  |
| sfHBeAg | Hepatitis B e Antigen (NcU/mL) at Follow-up Visit |  |
| sfHBeAb | Hepatitis B e Antibody (NcU/mL) at Follow-up Visit |  |
| sfHBcAb | Hepatitis B Core Antibody (NcU/mL) at Follow-up Visit |  |
| sfHBV DNA | HBV DNA (log10 IU/mL) at Follow-up Visit |  |
| sfWBC | White Blood Cell (10 ^12 /L) at Follow-up Visit |  |
| sfRBC | Red Blood Cell (10 ^12 /L) at Follow-up Visit |  |
| sfPLT | Platelet (10 ^9 /L) at Follow-up Visit |  |
| sfLYM | Lymphocyte (10 ^9 /L) at Follow-up Visit |  |
| sfAST | Aspartate Aminotransferase (U/L)at Follow-up Visit |  |
| sfALT | Alanine Aminotransferase (U/L) at Follow-up Visit |  |
| sfY-GT | Gamma-Glutamyl Transferase (U/L) at Follow-up Visit |  |
| sfTBIL | Total Bilirubin (umol /L)at Follow-up Visit |  |
| sfIBIL | Indirect Bilirubin (umol /L) at Follow-up Visit |  |
| sfTP | Total Protein (g/L) at Follow-up Visit |  |
| sfAlb | Albumin (g/L) at Follow-up Visit |  |
| tzHBsAg | Hepatitis B Surface Antigen (ng/mL) at IFN Discontinuation |  |
| tzHBsAb | Hepatitis B Surface Antibody(mIU/mL) at IFN Discontinuation |  |
| tzHBeAg | Hepatitis B e Antigen (NcU/mL) at IFN Discontinuation |  |
| tzHBeAb | Hepatitis B e Antibody (NcU/mL) at IFN Discontinuation |  |
| tzHBcAb | Hepatitis B Core Antibody (NcU/mL) at IFN Discontinuation |  |
| tzWBC | White Blood Cell (10 ^12 /L) at IFN Discontinuation |  |
| tzRBC | Red Blood Cell (10 ^12 /L) at IFN Discontinuation |  |
| tzPLT | Platelet (10 ^9 /L) at IFN Discontinuation |  |
| tzLYM | Lymphocyte (10 ^9 /L) at IFN Discontinuation |  |
| tzAST | Aspartate Aminotransferase (U/L)at IFN Discontinuation |  |
| tzALT | Alanine Aminotransferase (U/L) at IFN Discontinuation |  |
| tzY-GT | Gamma-Glutamyl Transferase (U/L) at IFN Discontinuation |  |
| tzTBIL | Total Bilirubin (umol /L)at IFN Discontinuation |  |
| tzIBIL | Indirect Bilirubin (umol /L) at IFN Discontinuation |  |
| tzTP | Total Protein (g/L) at IFN Discontinuation |  |
| tzAlb | Albumin (g/L) at IFN Discontinuation |  |
| STATE | Whether HBsAg-RS Occurs | 1=yes;0=no |
| TIME | Follow-up duration (days) |  |
| TIME2 | Follow-up duration (weeks) |  |
